# Critical changes in hypothalamic gene networks in response to pancreatic cancer as found by single-cell RNA sequencing

**DOI:** 10.1016/j.molmet.2022.101441

**Published:** 2022-01-11

**Authors:** Christian Huisman, Mason A. Norgard, Peter R. Levasseur, Stephanie M. Krasnow, Monique G.P. van der Wijst, Brennan Olson, Daniel L. Marks

**Affiliations:** 1 Papé Family Pediatric Research Institute, Oregon Health & Science University, Portland, United States; 2 Knight Cancer Institute, Oregon Health & Science University, Portland, United States; 3 Brenden-Colson Center for Pancreatic Care, Oregon Health & Science University, Portland, United States; 4 Department of Genetics, University of Groningen, University Medical Center Groningen, Groningen, the Netherlands; 5 Medical Scientist Training Program, Oregon Health & Science University, Portland, United States

**Keywords:** Cachexia models, Pancreatic cancer, Food intake regulation, Endothelial inflammation, scRNA-seq of the central nervous system, Neuroinflammation

## Abstract

**Objective:**

Cancer cachexia is a devastating chronic condition characterized by involuntary weight loss, muscle wasting, abnormal fat metabolism, anorexia, and fatigue. However, the molecular mechanisms underlying this syndrome remain poorly understood. In particular, the hypothalamus may play a central role in cachexia, given that it has direct access to peripheral signals because of its anatomical location and attenuated blood–brain barrier. Furthermore, this region has a critical role in regulating appetite and metabolism.

**Methods:**

To provide a detailed analysis of the hypothalamic response to cachexia, we performed single-cell RNA-seq combined with RNA-seq of the medial basal hypothalamus (MBH) in a mouse model for pancreatic cancer.

**Results:**

We found many cell type-specific changes, such as inflamed endothelial cells, stressed oligodendrocyes and both inflammatory and moderating microglia. *Lcn2*, a newly discovered hunger suppressing hormone, was the highest induced gene. Interestingly, cerebral treatment with LCN2 not only induced many of the observed molecular changes in cachexia but also affected gene expression in food-intake decreasing POMC neurons. In addition, we found that many of the cachexia-induced molecular changes found in the hypothalamus mimic those at the primary tumor site.

**Conclusion:**

Our data reveal that multiple cell types in the MBH are affected by tumor-derived factors or host factors that are induced by tumor growth, leading to a marked change in the microenvironment of neurons critical for behavioral, metabolic, and neuroendocrine outputs dysregulated during cachexia. The mechanistic insights provided in this study explain many of the clinical features of cachexia and will be useful for future therapeutic development.

## Highlights

• Single-cell RNA-sequencing reveals hypothalamic response to cancer cachexia.
• Tumor-derived factors induce endothelial inflammation in the hypothalamus.
• *Plin4* is a novel cachexia marker in oligodendrocytes for cancer cachexia.
• Novel hunger hormone LCN2 activates food-intake decreasing POMC neurons.
• Inflammatory and a moderating microglia in the hypothalamus respond to cancer cachexia.

## 1 Introduction

Cachexia is a wasting syndrome accompanying several chronic diseases, and it is characterized by involuntary weight loss, muscle wasting, abnormal fat metabolism, anorexia, and fatigue [1]. Although this syndrome is most common in pancreatic and gastric cancer, it occurs in many other types of cancers and chronic diseases such as rheumatoid arthritis and chronic renal failure. The underlying etiology of cachexia is multifactorial and is the result of crosstalk between different organs and the release of pro-inflammatory signals from affected peripheral tissues. The onset of cachexia has a profoundly negative impact on quality of life of affected individuals, and cancer cachexia patients have a low tolerance for cancer treatments, further exacerbating mortality. Therefore, a better understanding of the molecular mechanisms behind this condition is required to provide better treatment options that can improve the quality of life and prognosis of patients.

The initial physiological response of the body to inflammatory threats, known as acute illness response, is an evolutionarily conserved program that serves to neutralize immediate threats and is essential for survival. In contrast, cachexia can be considered as a manifestation of the unsustainable metabolic demands placed on the body due to persistent disease, and a large body of evidence suggests that this detrimental response is regulated by the brain [2,3]. In particular, the hypothalamus plays a central role in cachexia [4], given that it has direct access to peripheral signals due to its anatomical location and attenuated blood–brain barrier (BBB). Indeed, during cancer-cachexia, the hypothalamus responds inadequately to neuroendocrine signals, as exemplified by the dysregulation of appetite suppressing POMC neurons and appetite stimulating NPY/AgRP neurons [5]. Interestingly, aggressive subtypes of cancer and chronic renal failure are associated with the systemic induction of lipocalin-2 (*Lcn2*) [6], coding for a protein recently discovered to be involved in the regulation of appetite [7] and recently demonstrated by our group to play a critical role in appetite suppression during pancreatic cancer cachexia [8]. In addition, LCN2 can cross the BBB and bind to the hypothalamus [9], although it is unclear yet whether LCN2 affects food-regulating AgRP/POMC neurons.

Currently, little is known about the exact molecular mechanisms that drive chronic cachexia pathology in the medial basal hypothalamus (MBH), including the cell type-specific gene expression and gene networks. Only recently, the complexity of this region was revealed using single-cell RNA sequencing (scRNA-seq) [[10], [11], [12]], revealing more than 50 different distinct cell types. Each of these cell types could be contributing to the neuroendocrine, autonomic, and behavioral outputs found in cachexia. For example, microglia can produce high levels of IL-1β, IL-6, and TNFα, which in turn can affect food intake-regulating neuronal networks via NF-κB signaling [13], and even oligodendrocyte progenitors were found to respond to changes in food intake [14].

To increase our insight into the cell type-specific changes of hypothalamic cells under cachectic stress, we applied scRNA-seq using a syngeneic murine model of pancreatic ductal adenocarcinoma (PDAC) [15]. Our results reveal many cell type-specific changes, such as inflamed endothelial cells, stressed oligodendrocytes, and both inflammatory and moderating microglia. *Lcn2* was among the most highly upregulated transcripts in this region and using LCN2 treatment followed by RNA-seq, we found that LCN2 recapitulates key features of the cachectic phenotype, such as endothelial inflammation and oligodendrocyte stress. Interestingly, a subset of genes enriched in appetite suppressing POMC neurons as well as in appetite stimulating NPY/AgRP neurons were modulated upon LCN2 treatment, offering more insights for the decrease in food intake in cachectic mice. Re-analyses of scRNA-seq dataset of primary tumors (Steele et al., 2020) demonstrate that many changes found in the hypothalamus mirror those found in the tumor microenvironment (TME) itself, hinting that features of the local TME are recapitulated in the hypothalamus. Overall, our study provides comprehensive insights into the cell type-specific changes of hypothalamic cells under cachectic stress and provides several novel therapeutic targets to treat cancer cachexia.

## 2 Results

### 2.1 scRNA-seq of the MBH

**Figure 1:**
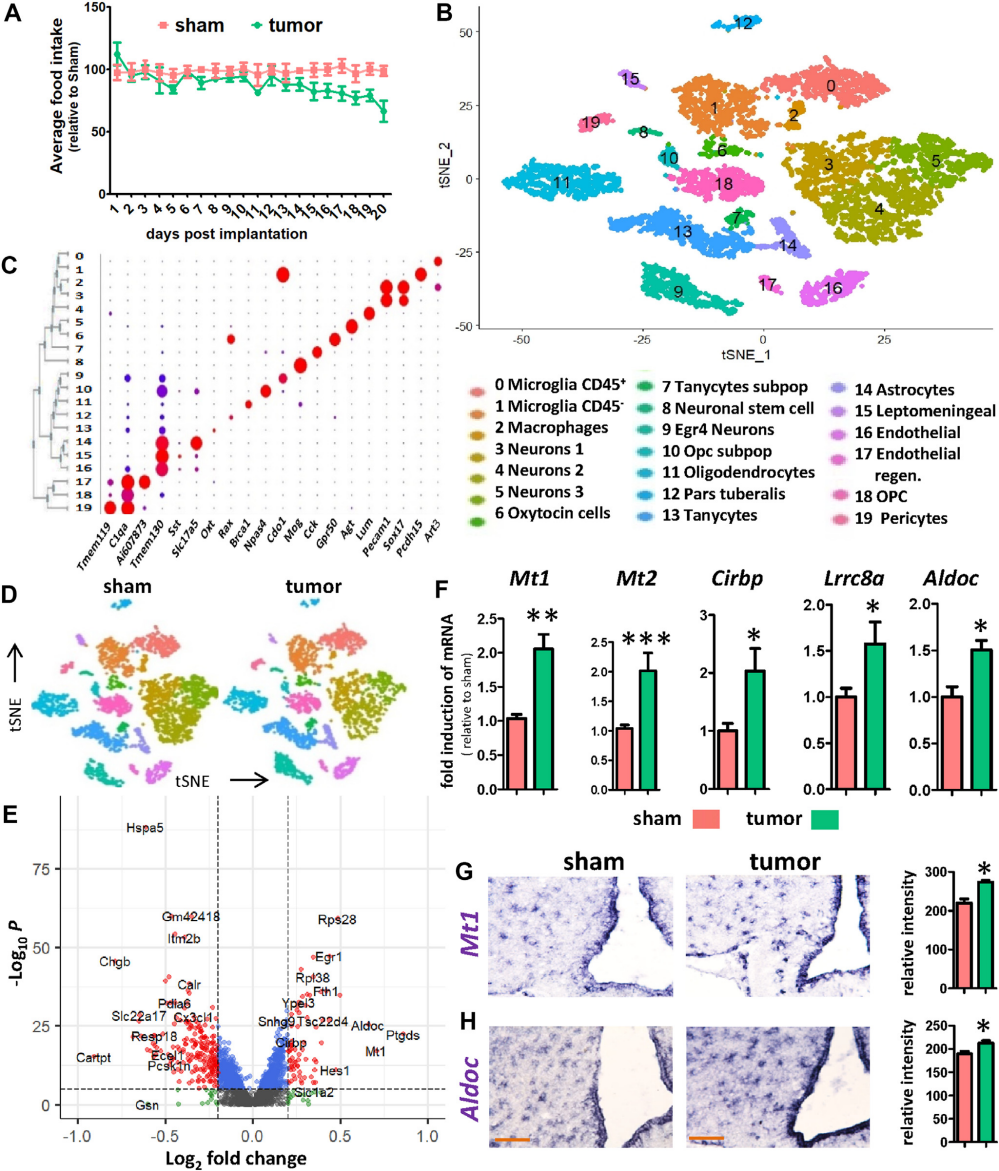
**scRNA-seq of the mediobasal hypothalamus reveals its responseto KPC-induced cachexia**. Food intake changes in KPC-induced cachexia over a period of 20 days post implantation (**A**). Unsupervised clustering of cells from sham and TB mice combined yields 20 different clusters as shown with a tSNE-plot (**B**). Classification of cell types was performed by identifying key marker genes. Dotplot showing the expression of cluster-specific genes (**C**). MBH cells from sham and TB mice show identical clustering pattern as shown with a tSNE plot (**D**). Differentially expressed (DE) genes between sham and TB mice in bulk as shown with a volcanoplot (**E**). qPCR analysis for stress related genes *Mt1, Mt2, Cirbp, Lrrc8a* and *Aldoc* (**F**). ISH for *Mt1* (**G**) and *Aldoc* (**H**) in the MBH for sham and TB mice and the quantification is shown on the right. Scale bar = 100 μm. Graph values represent the mean ± SEM of at least three independent experiments and statistical significance between groups was determined with a Student's t-test (∗P < 0.05, ∗∗P < 0.01, ∗∗∗P < 0.001).

To examine the effect of PDAC on hypothalamic gene expression, we selected KPC tumor cells for implantation in the pancreas. KPC cells are derived from a tumor of a C57BL/6 mouse modified to express oncogenic KRAS^G12D^ and the mutant P53^R172H^ allele under a pancreas-specific Pdx1-Cre driver [16]. Implantation of KPC cells resulted in the development of tumors exclusively in the pancreas, and food intake of the animals started to decrease ∼13 days after implantation (Figure 1A), indicating that the animals were in an early stage of cachexia. One week later, when the average food intake in the tumor-bearing (TB) mice was decreased by approximately 30%, 5 sham mice and 5 TB mice were used for scRNA-seq of the MBH (Figure S1A). The unsupervised clustering of 7136 cells (3854 cells obtained from sham and 3282 tumor cells obtained from TB mice) using Seurat identified 20 distinct clusters (Figure 1B). Subsequently, cluster specific markers were identified (Supplementary Table 2) and identification markers for the 20 clusters were visualized with a dot blot (Figure 1C), a heat map (Figure S1B), and featureplots (Figure S2), revealing identifiable astrocyte, tanycyte, endothelial, OPC, oligodendrocyte, 3 myeloid, and 4 neuronal clusters. Interestingly, of the 4 neuronal clusters, one cluster clearly separated from the other three neuronal clusters, and this cluster expressed specific transcripts (e.g. *Egr4* and *Npas4*). Using our classification, we could find novel marker genes for each cluster, such as *Mia* for tanycytes (Figure S1C). Myeloid clusters were identified by the expression of *C1qa*/*b*/*c* (Figure S1D) and other microglia markers (*Tyrobp*, *Trem2* (Figure S5A)).

To reveal those changes in the MBH that are affected in PDAC-associated cachexia, we assigned the combined cells from our initial clustering into a sham and TB mice group (Figure 1D), and every identified cell type contained cells from sham and TB mice (Figure S3A). Then, we determined the bulk differentially expressed (DE) genes and the cell type-specific DE genes (Supplementary Table 3) as well as the conserved genes (Supplementary Table 4) and plotted the number of DE genes per cluster (p < 0.005) (Figure S3B). We found that endothelial cells, oligodendrocytes, microglia, OPCs, and tanycytes were among the cell types, which were most affected by exposure to cachectic stress in our model. To define the degree of experimental noise in our samples, we compared DE genes with a published micro-array data set of cancer cachexia, where DE genes in the hypothalamus were determined using a Lewis lung carcinoma mouse model [5]. We found that from the 57 DE genes of the micro-array, 40 genes could be detected in the scRNA-seq samples and 68% of those could be confirmed in one of the cell types of our scRNA-seq (Figure S3C). To further confirm the quality of the single-cell data, we show with violin plots and featureplots that housekeeping genes, such as *Gapdh* and *Rpl13/14/15/17/18* show no difference between sham and TB mice (Figure S3D and S3E). *RPL13* was previously recommended as a reference gene for neuronal tissue in neurodegenerative disease [17]. Also, *Jun* expression was largely unchanged (Figure S3F), indicating that scRNAseq sample preparation did not lead to unwanted non-specific stress on the cells caused by cell dissociation.

### 2.2 Pancreatic tumors induce stress genes in the MBH

To find general changes across cell types during cachexia, we plotted the DE genes of the bulk scRNAseq using a volcano plot (Figure 1E) and violin plots (Figure S3D). Many of the highest induced DE genes are linked to cellular stress, such as *Sgk1*, *Rhob*, *Lrrc8a*, *Cirbp*, *Mt1*, *Mt2*, and *Aldoc*. More evidence for cellular stress in the MBH is the induction of *Hspa1a and Hspa1b*, which are heat shock proteins produced by cells in response to exposure to stressful conditions. The induction of *Lrrc8a* and *Cirbp* is indicative of hypotonic stress and genotoxic stress, respectively. Interestingly, *Cirbp* has been recently reported as a major novel gene regulating food intake using scRNA-seq [18]. Other highly induced genes are *Mt1* and *Mt2*, which were previously shown to mediate cachexia in skeletal muscle by altering zinc homeostasis [19]. Further evidence that the MBH is under stress is illustrated by the induction of *Aldoc*, an enzyme involved in anaerobic glycolysis, and its upregulation is indicative of cellular energy deficit, which may also occur under stress conditions. qPCR analysis confirmed that *Mt1*, *Mt2, Cirbp, Lrrc8a* and *Aldoc* are upregulated under cachectic stress (Figure 1F)(. Induction of *Aldoc* and *Mt1* in TB mice was further confirmed using in situ hybridization (ISH) (Figure 1G and H) and both genes were highly enriched in tanycytes. In addition to genes involved in stress responses, we found increased expression of numerous genes involved in small molecule transport, such as *Fth1*, an enzyme involved in iron homeostasis. Among the most highly downregulated genes are *Cartpt*, a gene involved in feeding behavior and *Hspa5*, a gene often downregulated under stress conditions [20].

**Figure 2:**
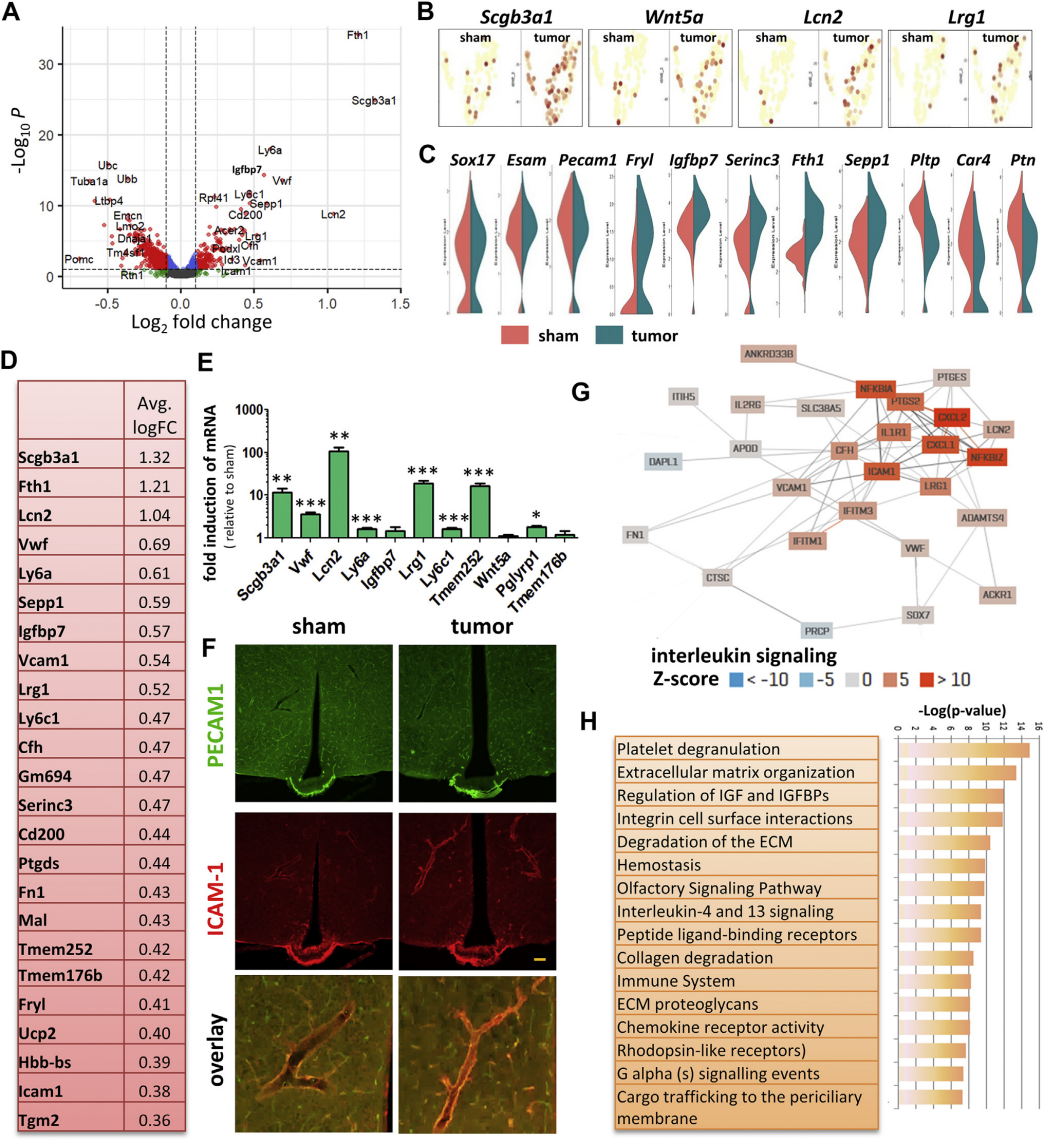
**MBH endothelial cell response to pancreatic cancer**. Differentially expressed (DE) genes between sham and TB mice in endothelial cells as shown with a volcano plot (**A**). Featureplots showing the induction of genes in TB mice linked to the immune system (*Lcn2*, *Lrg1*), membrane organization (*Scgb3a1*), as well as secreted protein *Wnt5a* (**B**). Vln plots showing examples of highly upregulated DE genes, highly downregulated DE genes as well as conserved genes between sham and TB mice in the endothelial cluster (**C**). Top 24 upregulated genes in endothelial cells in TB mice sorted by fold inductions (**D**). qPCR analysis of top-induced genes using whole hypothalamic RNA extracts from sham and TB mice (**E**). IHC for ICAM-1 and PECAM1 in the MBH for sham and TB mice (**F**). PECAM1 labels all endothelial cells, while ICAM-1 is only expressed in a subset of the endothelial cells. Co-expression network using the top-induced genes in *Lcn2*^+^ endothelial cells revealing a network linked to inflammation. Z-scores indicate REACTOME pathway enrichment for interleukin signaling (**G**). Reactome pathway analysis of top 60 induced genes in endothelial cells of TB mice (**H**). qPCR values represent the mean ± SEM of at least three independent experiments and statistical significance between groups was determined with a Student's t-test (∗P < 0.05, ∗∗P < 0.01, ∗∗∗P < 0.001). Scale bar = 100 μm.

### 2.3 Endothelial inflammation in the MBH

Because endothelial activation is an important mediator of hypothalamic inflammation, altered signaling in neurons, and general sickness responses, we undertook cell type-specific analyses of DE genes in endothelial cells. Robust changes in TB mice were detected as shown with a volcano plot, violin plots, and/or featureplots (Figure 2A–C). The significantly changed genes included several inflammation mediators (*Vwf*, *Lcn2*, *Lrg1*), genes that stimulate adhesion (*Igfbp7*, *Icam1*, *Fn1*), transmembrane proteins (*Tmem176a*, *Tmem176b*, *Tmem252*), genes involved in iron uptake and release (*Ftn1*, *Lcn2*) and antigen presenting genes (*Ly6c1*, *Ly6a*) (Figure 2D). No changes were detected in known endothelial marker genes like *Esam*, *Pecam1* and *Sox19*. Among the most significantly downregulated genes were *Car4* and *Ptn*. The gene with the greatest induction was *Scgb3a1*, a secreted cytokine that is involved in cell surface tension, immunoregulatory perturbations [21] and BBB dysfunction during disease [22]. Although the function of some of these genes has not been individually described, constructing a co-expression plot can predict some of their functions in complex tissues, as shown for *Tmem252* (Figure S4A); which is predicted to link three separate networks controlling either nitric oxide release, transport of bile salts or Ncam interactions. qPCR analysis confirmed upregulation of many of the DE genes in endothelial cells, and relative induction of *Lcn2* was highest (183 ± 42 fold) among the measured genes (Figure 2E). *Icam1*, a gene known to regulate vascular permeability and inflammation [23] was further analyzed by IHC (Figure 2F). Interestingly, large blood vessels surrounding the arcuate nucleus drastically increase ICAM1 expression, while endothelial PECAM1 remained unchanged between sham and TB mice. IHC analysis of VWF and LCN2 show they are co-expressed in the blood vessels supplying the MBH in TB mice (Figure S4B). We also confirmed the induction of *Sepp1* by ISH, a secreted selenoprotein, which acts as an extracellular antioxidant (Figure S4B).

To reveal potential interplay between induced genes in affected endothelial cells, we visualized the co-regulation network in affected endothelial cells followed by pathway analysis (Figure 2G and H). The resulting gene network was primarily linked to inflammation and consisted of genes that are part of the innate immune system, extracellular matrix (ECM) remodeling, integrin/adhesion regulation, platelet degranulation, and interleukin signaling. Interestingly, the highest induced gene *Lcn2* is known to be an important contributor to cachexia. However, the factors responsible for *Lcn2* induction and its downstream effects are unknown, but LCN2 may contribute to cancer anorexia via its action on hypothalamic melanocortin signaling [7].

### 2.4 Two types of microglia found in the MBH respond differently to KPC-induced cachexia

**Figure 3:**
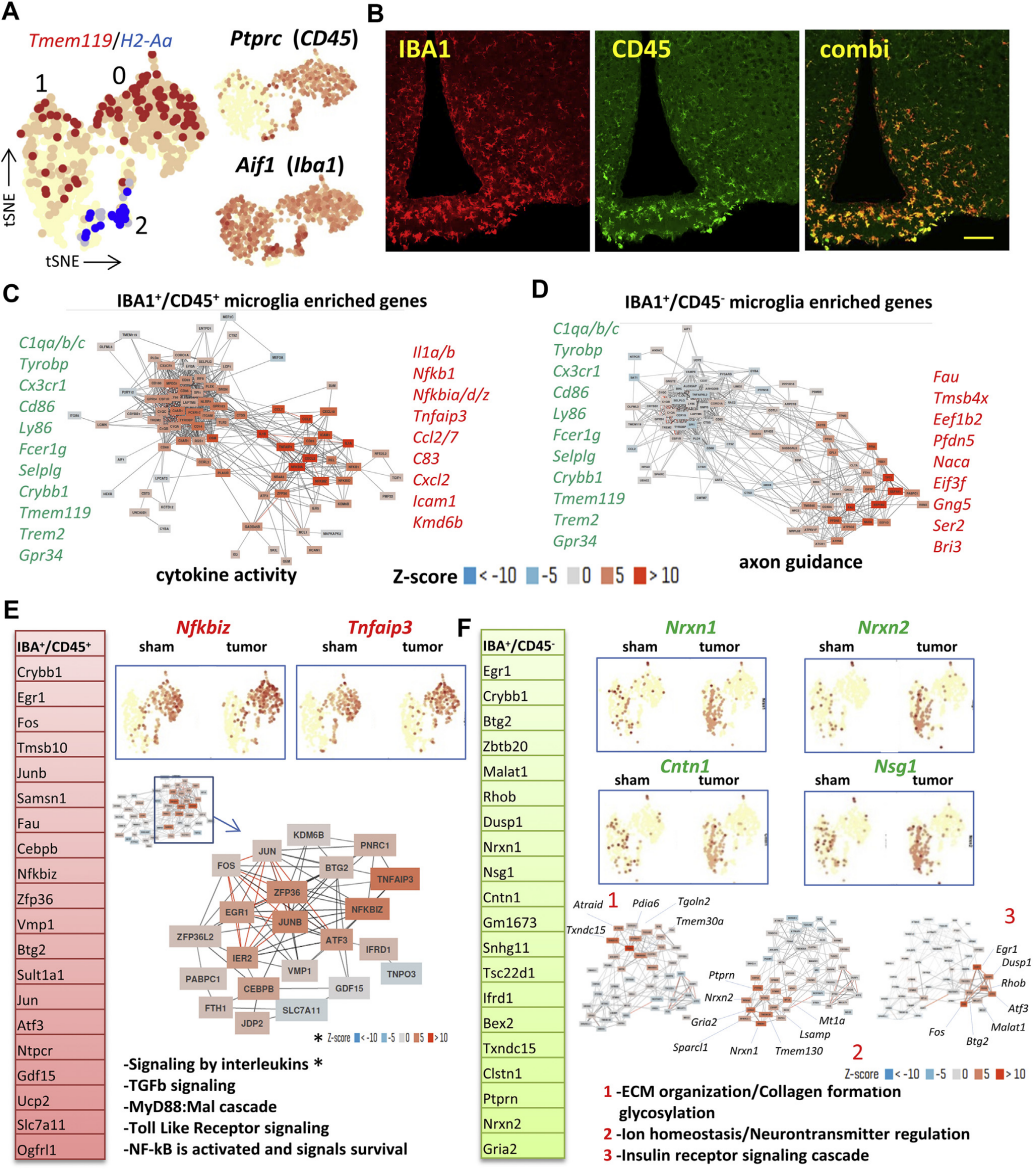
**Two types of microglia found in the MBH respond differently to KPC-induced cachexia**. Featureplots showing the expression of microglia specific *Tmem119*, macrophage specific *H2-Aa*, as well as *Ptprc* (*Cd45*) and *Aif1* (*Iba1*) in the myeloid cells-containing clusters (**A**). Visualization of IBA1^+^/CD45^+^ microglia and IBA1^+^/CD45^−^ negative microglia in the MBH as observed with IHC (**B)**. Co-regulation network of the top 100 enriched genes (from Table S2) in IBA^+^/CD45^+^ microglia (sham and TB mice combined) reveals a network of core-microglia genes as well as a network of genes mostly linked to cytokine activity (**C**). Similarly, clustering for IBA^+^/CD45^−^ cells reveals the cluster of core-microglia genes, as well as a cluster of genes mostly linked to axon guidance. Ribosomal genes were removed before analysis (**D**). Top-induced genes in IBA^+^/CD45^+^ microglia in TB mice (from Table S3) and featureplots are shown for *Nfkbiz* and *Tnfaip3* for the myeloid clusters. Also shown is the activated gene network in IBA^+^/CD45^+^ cells in TB mice as observed with unbiased clustering and the pathways associated with this gene network (**E**). Similarly, for IBA^+^/CD45^−^ the top-induced genes are shown, as well as examples with featureplots for *Nrxn1*, *Nrxn2*, *Cntn1* and *Nsg1*. Also shown are the activated gene network in IBA^+^/CD45^−^ as observed with unbiased clustering consisting of three separated gene clusters each with its own associated pathways (**F**). Scale bar = 100 μm.

Next, we assessed the changes in the two microglia clusters, as well as in the closely related macrophages. We found two separate clusters of microglia cells in the MBH. Both microglia clusters were positive for the microglial markers *Tmem119* and *Iba1*, but *Cd45* was only expressed in one of the microglial clusters (Figure 3A). To confirm these findings, we visualized both CD45 and IBA1 in the MBH (Figure 3B), and found that the IBA1^+^^/^CD45^+^ cells are located adjacent to the third ventricle, while the IBA1^+^^/^CD45^−^ microglial cells are located more dorsal and lateral to the third ventricle. While the IBA1^+^/CD45^+^ were mostly positive for conventional microglia markers like *P2ry12*, *Cx3cr1* and *Tnf* (Figure S5B), the IBA1^+^/CD45^−^ population was enriched for actin, ribosomal and iron related genes, and therefore are consistent with immature microglia [24] (Figure S5C). The third myeloid population was identified as macrophages by the expression of the antigen presenting marker H2-Aa (Figure 3A). Co-expression networks of enriched genes in IBA1^+^/CD45^+^ and IBA1^+^/CD45^−^ subtypes reveals two distinct gene networks in both types of microglia. While both subtypes display a network of constitutively expressed microglia genes, such as C1qa/b/c, *Ly86* and *Tyrobp*, the second network was linked to either cytokine activity for IBA1^+^/CD45^+^ microglia or axon guidance for IBA1^+^/CD45^−^ microglia (Figure 3C and D). Interestingly, IBA1^+^/CD45^+^ microglia responded differently to the induced cancer cachexia than IBA1^+^/CD45^−^ microglia. Co-expression networks followed by pathway analysis of the top 50 induced transcripts demonstrated that IBA1^+^/CD45^+^ microglia activated pathways traditionally linked to inflammation, such as interleukin and cytokine signaling, Toll-like receptor cascades and MyD88:Mal cascades, with induction of genes like *Vmp1*, *Samsn1*, *Nfkbiz*, and *TNFAip3* (Figure 3E). In contrast, gene clustering followed by pathway analysis of the top 50 induced genes in IBA1^+^/CD45^−^ microglia revealed the induction of three distinct gene clusters, with associated pathways linked to either ECM regulation, ion homeostasis or insulin signaling (Figure 3F, Figure S6), and the induction of genes such as *Nrxn1*, *Nrxn2*, *Nsg1*, and *Cntn1* (Figure 3F). qPCR confirmed the significant induction of *Nfkbiz*, *Tnfaip3*, and *Crybb1* (Figure S5D), while induction of other tested microglia-enriched genes in the MBH, such as *Gdf15* and *Slc7a11* was not significant, indicating weak microglial activation. However, using *Myd88* KO mice, we could prevent the activation of the IBA1^+^/CD45^+^ microglia, as demonstrated by the downregulation of *Nfkbiz* in these TB mice (Figure S5E). *Crybb1*, a gene implicated in synaptic pruning [25], anxiety behavior and stress [26], was upregulated in both microglia clusters (Figure S5F). We also observed a modest induction of microglia activation marker IBA1 using IHC (Figure S5G), as we have previously published [27], but this induction was not observed at the RNA level (Figure S5D and S5F).

Macrophages displayed increased signaling by the TGF-beta receptor complex (Figure S5H), with the induction of genes such as the chemokine receptor *Ccr1* (Figure S5i) and the inflammatory cytokine *Il1a* (Supplementary Table 3). However, the number of detected macrophages in our samples was low (∼15–20 per condition) and heterogeneous.

### 2.5 Oligondendrocytes respond to cachectic stress

**Figure 4:**
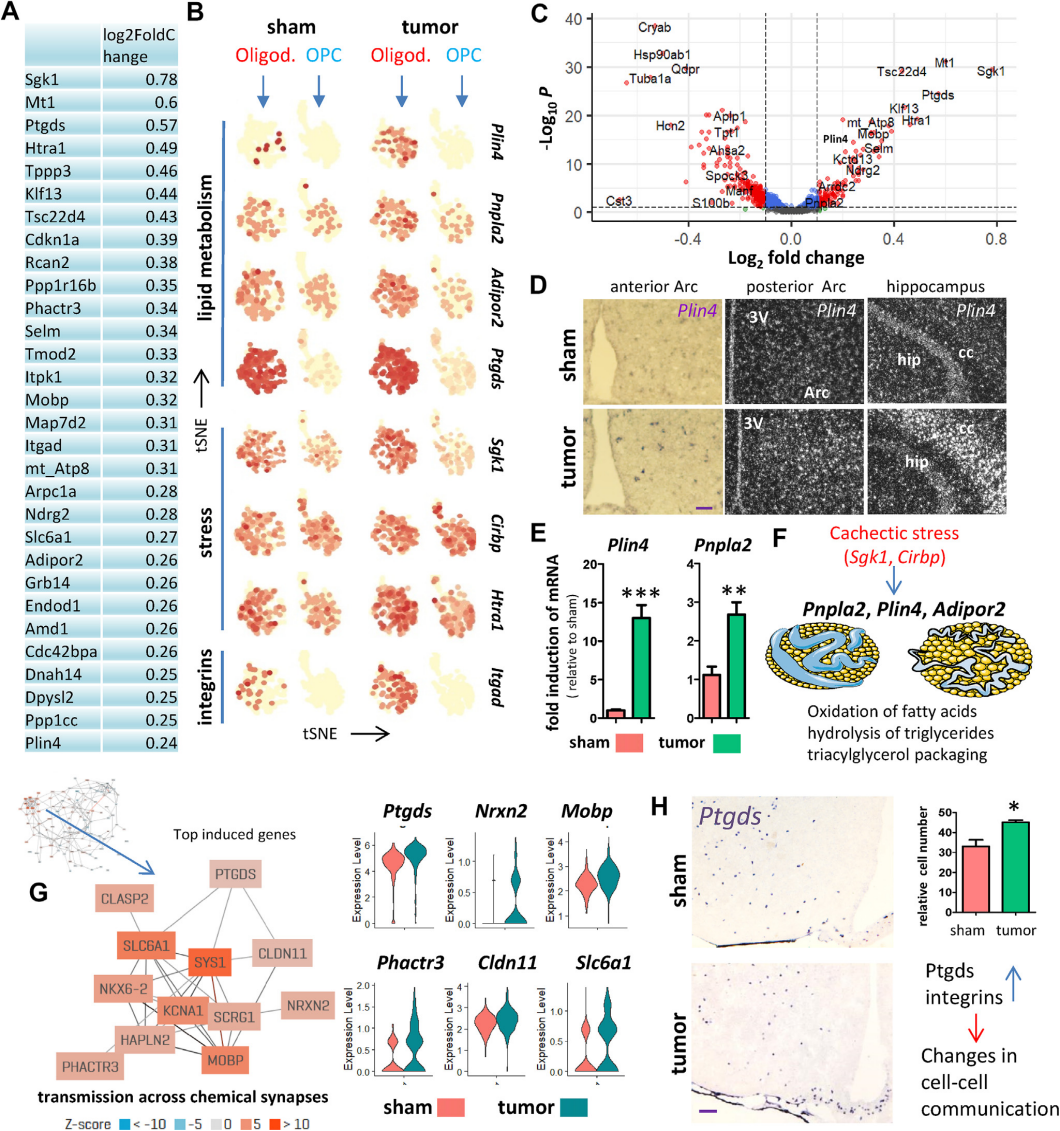
**MBH oligodendrocyte response to pancreatic cancer**. Top-induced genes in oligodendrocytes in TB mice ranked by fold inductions (**A**). Featureplots showing the induction of genes in oligodendrocytes linked to fatty acid synthesis, stress and cell adhesion (**B**). DE genes between sham and TB mice in oligodendrocytes as shown with a volcano plot (**C**). ISH against *Plin4* reveals increased *Plin4* expression in brain sections of TB mice compared to sham (**D**). qPCR analysis for *Plin4* and *Pnpla2* using whole hypothalamic RNA extracts from sham and TB mice (**E**). Proposed model whereby the stress-responsive genes *Sgk1* and *Cirbp* are proposed to act as master regulators of the cachexia response in oligodendrocytes by regulating genes linked to catabolism of fatty acids and starvation conditions (**F**). Co-expression network using the top-induced genes in oligodendrocytes revealing a network linked to transmission across chemical synapses. The subnetwork of co-regulated genes is highlighted, and violin plots on the right showing the expression of network members for sham and TB mice (**G**). ISH against *Ptgds* reveals increased expression in brain sections of TB mice compared to sham, also shown is the quantification (**H**). The increase in *Ptgds* and integrins is proposed to impact communication between cell types in the MBH. Scale bar = 100 μm. Graph values represent the mean ± SEM of at least three independent experiments and statistical significance between groups was determined with a Student's t-test (∗P < 0.05, ∗∗P < 0.01, ∗∗∗P < 0.001).

In our cachexia mouse model, several of the DE genes enriched in the bulk analysis (Figure 1E, Supplementary Table 3) belong to the top-induced genes in oligodendrocytes (Figure 4A–C), such as *Mt1*, *Sgk1*, *Klf13*, and *Ptgds*, indicating an important role for oligodendrocytes in the pathology of cachexia. *Plin4*, *Ptgds*, and *Itgad* are among the DE genes more specific for oligodendrocytes, and induction of *Plin4* was confirmed with ISH and qPCR (Figure 4D and E). *Plin4* induction was not confined to the MBH, but rather was increased widely throughout the brain. Several of the DE genes in oligodendrocytes are linked to lipid metabolism (Figure 4B), such as *Pnpla2*, and induction of this gene in TB mice was confirmed with qPCR (Figure 4E). Activation of genes involved in lipolysis may be the consequence of cachectic stress and the limited availability of nutrients (Figure 4F) which would be expected to worsen the outcome of cachexia. Clustering of the top-induced genes in oligodendrocytes reveals a subnetwork of genes co-regulated by *Ptgds* (Figure 4G), one of the highest induced genes in this cell type. The induced gene network is predicted to function in transmission across chemical synapses and violin plots demonstrate the induction of the individual genes of this network (Figure 4G). Indeed, one of the predicted functions of *Ptgds* is regulating neurotransmitter activity (Figure S7). We then confirmed by ISH that *Ptgds* is induced in TB mice and that its expression is confined to oligodendrocytes and leptomeningeal cells as predicted by scRNA-seq (Figure 4H). Given its function to control chemical transmission, we predict that oligodendrocytes alter cell–cell communication in the hypothalamus when placed under cachectic stress.

### 2.6 Modulating LCN2 expression in the hypothalamus

**Figure 5:**
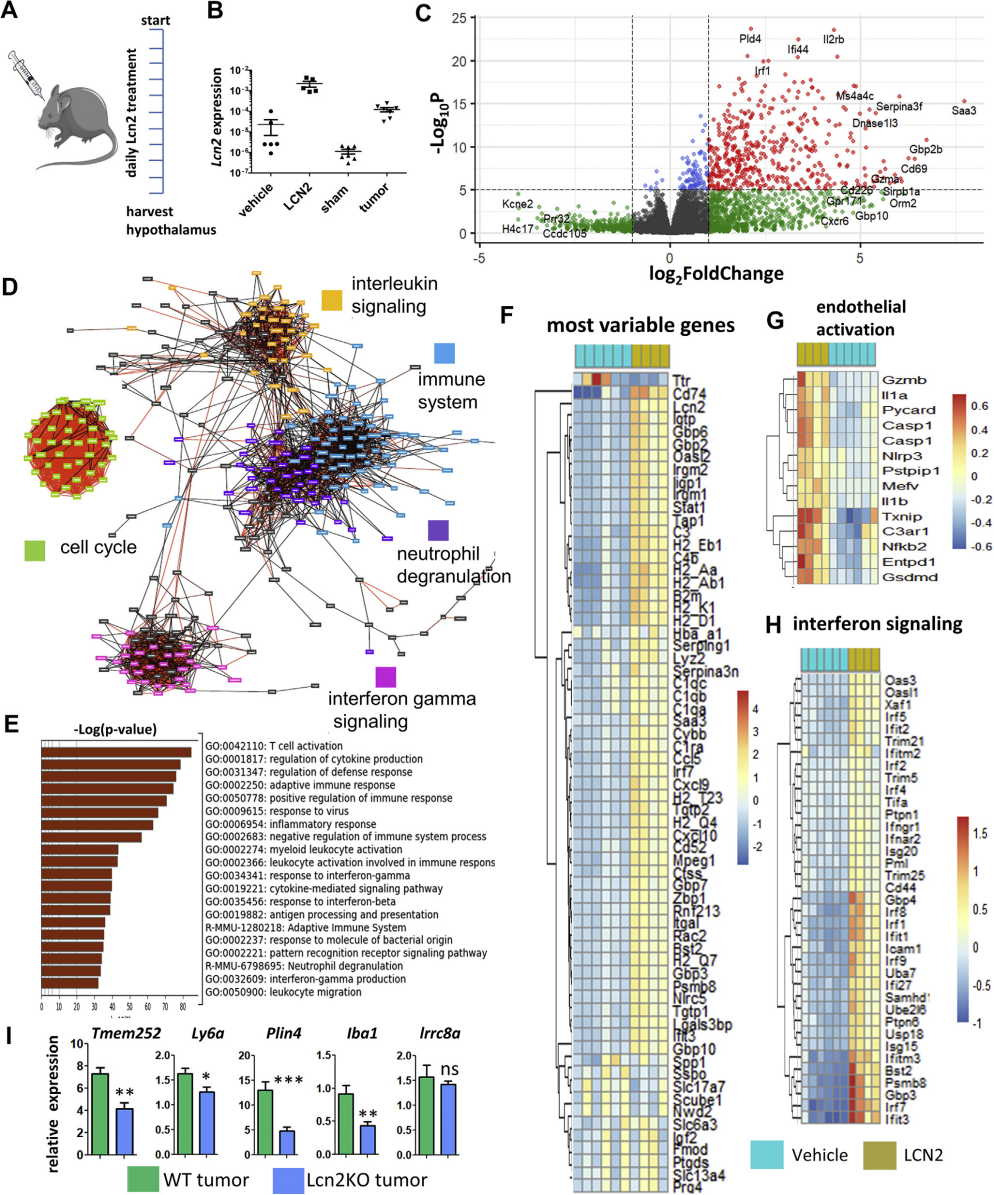
**LCN2 treatment induces an immune response in the hypothalamus**. Treatment schedule of mice receiving LCN2 or vehicle every morning through a lateral ventricle cannula for 10 days (**A**). Expression of *Lcn2* in LCN2-treated mice was confirmed with qPCR and compared with sham and TB mice (**B**). Differential gene expression assessed by RNA-seq in the hypothalamus of LCN2 or vehicle-treated mice as shown with a volcano plot (**C**). Main gene expression networks activated after LCN2 treatment as found by RNA-seq using the top-induced genes (**D**). Pathway analysis performed on genes with increased expression after LCN2 treatment, as analyzed with metascape on the top 1000 induced genes from the RNA-seq (**E**). Top-65 variable genes after LCN2 treatment as shown with a heatmap (**F**). Heatmap for genes involved in endothelial activation (**G**) and interferon signaling (**H**) after LCN2 treatment as found by RNA-Seq. qPCR analysis of cachexia markers for WT and Lcn2 KO TB mice (**I**). qPCR values represent the mean ± SEM of at least three independent experiments and statistical significance between groups was determined with a Student's t-test (∗P < 0.05, ∗∗P < 0.01, ∗∗∗P < 0.001).

*Lcn2* was among the most highly upregulated transcripts in the hypothalamus and was recently implicated in the suppression of food intake [7], one of the key symptoms of cachexia, but its exact function is still unknown. To obtain a more complete picture of the function of LCN2 in the hypothalamus, we treated WT mice via intracerebroventricular injection with LCN2 or vehicle (Figure 5A and B) and performed RNA-seq of the hypothalamus and visualized the changes with a volcano plot (Figure 5C), gene networks (Figure 5D), and heatmaps (Figure 5F, Figure S8). After LCN2 treatment, the most striking observation is a gene signature consistent with the activation of the immune system and induction of inflammation as illustrated, with three major immune gene networks activated upon LCN2 treatment. These are linked to interleukin signaling, neutrophil degranulation, and IFG signaling. A fourth observed network was linked to cell cycle regulation (Figure 5D). Pathway analysis of the top 1000 induced genes (Supplementary Table 5) revealed T cell activation, cytokine signaling and regulation of a defense response as the top 3 pathways (Figure 5E). Interestingly, LCN2 did not induce a stress response in the hypothalamus as seen in TB-mice (data not shown). The most differentially expressed downregulated gene was *Ttr*, a carrier protein involved in the transportation of thyroid hormone. To get a better idea of which of the identified cachexia genes found with the scRNAseq are caused by LCN2 induction, we generated heatmaps of those differentially expressed genes from the LCN2 RNA-seq data for endothelial cells, microglia and oligodendrocytes (Figure S8A). This data shows that LCN2 could be responsible for many of the upregulated genes found in endothelial cells in the hypothalamus under cachectic stress, as well as for some of the oligodendrocyte genes, such as *Plin4*, *Sgk1* and *Adipor2*. In accordance with this observation that LCN2 activates the immune system, LCN2 induced the expression of several microglia genes in IBA1^+^/CD45^+^ microglia, such as *Crybb1*, *Slc7a11*, *Junb* and *Ucp2*, and to a lesser extent in IBA1^+^/CD45^−^ negative microglia, although induction of *Crybb1* was very prominent as well in this microglia population. qPCR confirmed induction of endothelial inflammation genes, microglia genes and cachexia specific oligodendrocyte genes after treatment with LCN2, while stress genes *Lrrc8a*, *Mt1* and *Cirbp* remained unaffected (Figure S8B).

To find more proof that LCN2 primarily affects endothelial cells, oligodendrocytes, and immune cells in the MBH under cachectic conditions, we used TB Lcn2 KO mice to determine if removal of *Lcn2* affects our cachexia marker genes in these cell populations. First, we found that Lcn2 KO mice had more moderate cachexia and correspondingly decreased induction of a subset of the DE genes found in our scRNAseq model (Figure 5I), such as *Plin4*, *Tmem252*, and *Ly6*a.

### 2.7 Cell–cell communication in the MBH

**Figure 6:**
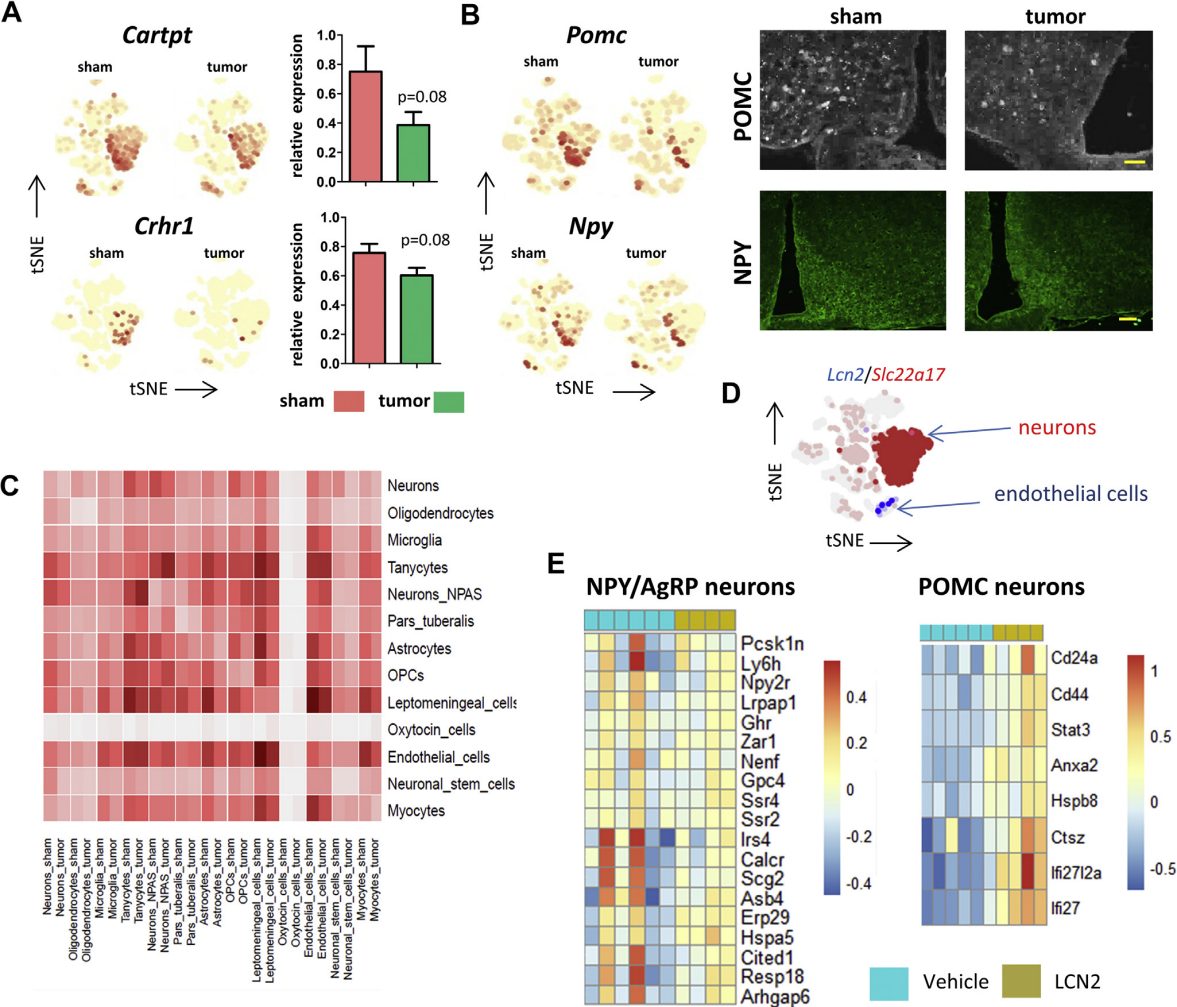
**Cell–cell communications in the MBH**. Featureplots for *Cartpt* and *Crhr1* for sham and TB mice as well as quantification by qPCR (**A**). Featureplots for anorexigenic *Pomc* and orexigenic *Npy* for sham and TB mice, as well as IHC for POMC and NPY in the MBH for sham and TB mice (**B**). Heatmap of the total number of ligand–receptor interactions between sham and TB mice. Each cell type is compared to cell types of the same condition (sham left, TB mice right) as found by scRNA-seq (**C**). Example of a potential cell–cell interaction in cancer cachexia with the receptor indicated in red and the ligand in blue (**D**). Heatmap for genes enriched in appetite stimulating NPY/AgRP neurons which are downregulated upon LCN2 treatment (left) and heatmap for genes enriched in appetite-decreasing POMC neurons which are upregulated after LCN2 treatment (right) (**E**). Enriched genes for NPY/AgRP neurons or POMC neurons were obtained by scRNA-seq using the top 100 enriched genes of the both subtypes of neurons as found by Campbell et al. 2017 [10]. qPCR values represent the mean ± SEM of at least three independent experiments and statistical significance between groups was determined with a Student's. Scale bar = 100 μm.

The impact of cachectic stress on the interaction between the various cell-types in the hypothalamus likely affects neuronal functioning, contributing to observed behavioral and metabolic alterations typical of cachexia. Indeed, previous studies demonstrate that hypothalamic neuronal peptides regulating food intake are modulated in cancer cachexia [15], and the scRNAseq data indicate that the anorexigenic *Pomc*, *Cartpt*, and *Crhr1* were among the most downregulated transcripts in neurons in TB mice, while the orexogenic *Npy* showed little change (Figure 6A and B). We validated the downregulation of POMC by IHC, and show that NPY levels remain unchanged in TB mice (Figure 6B). However, the downregulation of *Cartpt* and *Crhr1* by qPCR did not reach significance. The induced changes are likely the result of crosstalk between the different cell types in the MBH and brain areas that provide inputs to this region, as well as being secondary to changes in peripheral signals of overall metabolic status.

To establish meaningful interactions between the various cell types, we used CellPhoneDB [28] for the identification of potential ligand–receptor interactions between the clusters (Figure 6C), and listed all identified interactions for sham and TB mice (Supplementary Table 6). Using this tool, we found that tanycytes, endothelial cells, and leptomeningeal cells show the highest potential interaction with other cell types, while oligodendrocytes, neuronal stem cells, and oxytocin cells show only few potential interactions. Furthermore, tanycytes, endothelial cells, OPCs, as well as *Npas4* neurons show a potential increase in interactions under cachectic stress. Of interest for this study is the ligand–receptor interactions of *Lcn2* with its putative neuronal receptor *Slc22a17* [29] on neurons as plotted in Figure 6D, and we hypothesized that overexpression of LCN2 may affect neuronal genes involved in feeding. Indeed, a recent study has shown that LCN2 is an anorexigenic signal in primates and has hypothalamic binding sites [9]. To find out which genes are modulated in food-intake regulating neurons in the hypothalamus, we obtained the top 100 enriched genes of either orexigenic AgRP neurons as well as the the top 100 enriched genes in anorexigenic POMC neurons using previously published scRNA-seq data of the hypothalamus [10]. We then generated heat maps for those genes which are de-activated in AgRP neurons as well as for the genes that are activated in POMC neurons after LCN2 treatment (Figure 6E). This data show that expression of several NPY/AgRP enriched genes, such as *Irs4*, *Calcr*, *Scg2*, *Cited1*, and *Resp18*, is higher in 2 of the 5 vehicle treated animals compared to LCN2 treated animals, however, no significance was reached. In contrast, LCN2 treatment significantly enhanced expression of POMC-enriched genes *Cd24a*, *Cd44*, *Stat3*, *Anxa2*, *Hspb8*, *Ctsz*, *Ifi2712a*, and *Ifi27*. These data may indicate that LCN2 directly affects gene expression in these food-intake regulating neurons of the arcuate nucleus.

### 2.8 Cachexia markers in the TME

**Figure 7:**
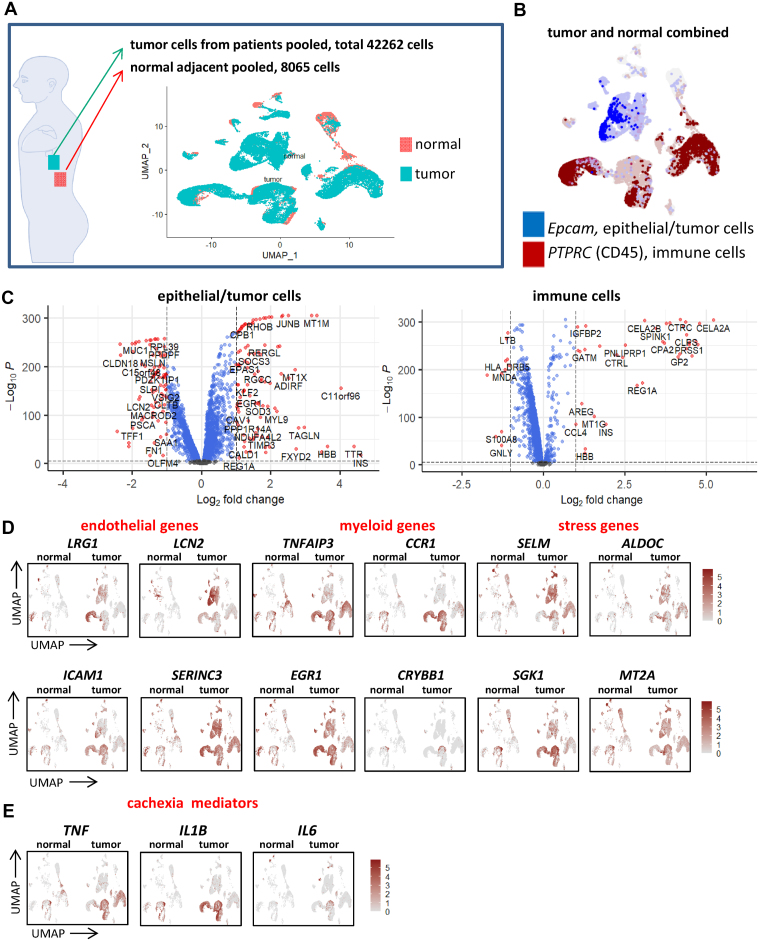
**Expression of cachexia markers at the primary tumor site**. Re-analysis of Steele et al.'s single cell dataset (Steele et al., 2020) of 16 samples isolated from human PDA patients and 3 adjacent normal pancreas samples (**A**). All normal and tumor cells combined can be roughly divided into two groups, either neoplastic cells (*Epcam*^+^) or immune cells (CD45^+^) (**B**). Differential gene expression in epithelial/tumor cells (left) and immune cells (right) between pancreatic tumor cells and normal pancreatic cells as obtained from the single cell dataset visualized with volcano plots (**C**). Featureplots showing the induction of genes in pancreatic tumors linked to endothelial inflammation, myeloid cells and cell stress (**D**). Featureplots showing abundant expression of cachexia mediators in pancreatic tumors (**E**).

A possible explanation for some of the detected cachectic responses in the MBH is that they originally arise in the TME itself, and subsequently transferred via mediators to the MBH to control behavior. To this end, we re-analyzed scRNA-seq data obtained from pancreatic cancer patients and control samples from a previously published study (GSE155698) [30] for the expression of our cachexia markers and possible mediators. A total of 42262 cells isolated from 16 PDA patients and 8065 cells from 3 normal adjacent pancreas samples were re-analyzed (Figure 7A) revealing *Epcam*^*+*^ tumor cells and CD45^+^ immune cells (Figure 7B). Then, we determined the main differences between tumor- and normal pancreas samples for tumor cells and immune cells and visualized these using volcano plots (Figure 7C). Remarkably, many of these genes modulated in the MBH under cachectic stress are induced in the TME as well. For example, among the top 10 induced genes in the tumor compartment are 4 members of the metallothionein family of genes (MT1A, MT1M, MT1X and MT2A), while secreted protein IGFBP7 was ranked number 15. Most of the genes in the top 250 were either linked to ECM organization, interleukin signaling or digestion (data not shown). Similarly, for the immune cell population 5 members of the metallothionein family of genes were upregulated in the top 100 of induced genes (MT1E/G/H/M/X). To look more specifically at the main cachexia markers that we found in the MBH, we visualized endothelial, myeloid, and stress genes using feature plots for the tumor samples and controls (Figure 7D). Indeed, these cachexia markers in the MBH are highly expressed in the tumors of PDA patients. Interestingly, many CCR1+ macrophages accumulate at the TME (Figure 7D), and this is a possible source for the enhanced CCR1+ macrophages found in the MBH under cachectic stress. These CCR1+ macrophages expressed cachexia mediators *Tnf*, *Il1b*, and *Il6* (Figure 7E).

## 3 Discussion

**Figure 8:**
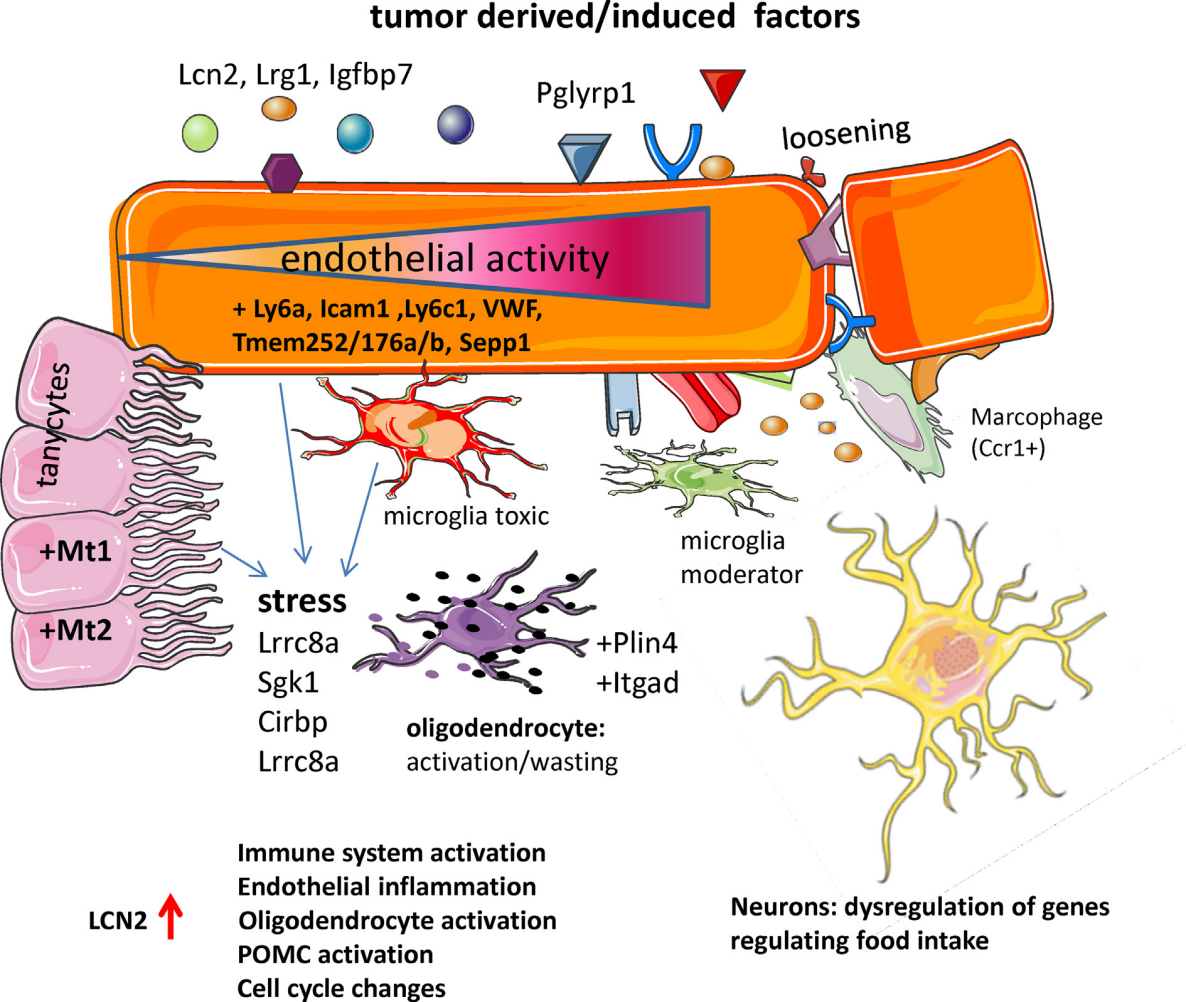
**Model for cachexia-induced changes in the MBH**. Tumor-derived factors cause local stress on endothelial cells affecting many features of endothelial functioning such as expression of adhesion molecules and transmembrane molecules, as well as loosening of the BBB. Tanycytes, which line the ventricle walls, increase expression of metallothioneins. Microglia may either be protective or neurotoxic and *Ccr1*^+^ macrophages are enriched in the cachectic MBH. The altered state of the BBB affects other cell types such as the oligodendrocytes, which upregulate stress and adhesion molecules. LCN2 activates many features of the immune system, but also other cell types are affected. The combined actions of these cell types ultimately influences neuronal functioning, which display disturbed expression of genes involved in food intake.

Previous studies demonstrated the role of MBH as one of the drivers of metabolic disturbances in cancer cachexia, but the molecular mechanisms underlying this syndrome are not well understood. Here, we investigated for the first time the molecular changes at a cellular level and provide several important insights into the development of cancer cachexia (Figure 8). Our scRNA-seq analysis reveals that multiple cell types in the MBH are affected by tumor-derived factors or host factors that are induced by tumor growth, leading to a marked change in the microenvironment of neurons critical for behavioral, metabolic, and neuroendocrine outputs dysregulated during cachexia. For example, profound changes were found in endothelial cells, which have proximal access to circulating factors from peripheral tissues. They displayed altered expression of membrane proteins, adhesion molecules and a breakdown of the ECM, leading to a loss of membrane integrity and loosening of the BBB. We also found two types of microglia with distinct response patterns: the IBA^+^/CD45^+^ microglia demonstrated primarily a traditional inflammatory response, whereas the IBA^+^/CD45^−^ cells had a moderating function, consistent with our prior observations [27]. We also hypothesized that cellular energy depletion contributed to the observed pathological response in oligodendrocytes, causing further harm to neuronal function. We show that *Lcn2*, the highest induced gene, has an important role in mediating various features of cachexia, and our RNA-seq precisely depicts its role in endothelial inflammation and activation of various components of the immune system. Moreover, the RNA-seq data indicated that LCN2 affected gene expression in anorexigenic POMC neurons, which may have relevance in cachexia-anorexia, although POMC expression itself was unchanged. Similarly, the scRNA-seq data indicates that the anorexic phenotype of the TB mice are independent of POMC expression. Given that anorexia in this model is reversible with melanocortin antagonists [8,31], this lends further support to the idea that normal homeostatic melanocortin signaling is bypassed by LCN2 acting as an MC4R agonist under pathological conditions. Food-intake regulation is a complex process influenced by numerous metabolic, physiological, and behavioral factors, and it is also likely that the general state of stress in the MBH during cancer cachexia overrides the normal physiological function of individual genes orchestrating behavioral output.

One limitation of this study is that we cannot distinguish the specific impact of each feature of this complex syndrome (e.g. weight loss, decreased food intake, etc.) on the observed change in hypothalamic transcriptome. However, the remarkable overlap in the behavioral and transcriptomic response to cancer and to LCN2 administration serves to strengthen the connection between hypothalamic responses and systemic inflammation. LCN2 was originally described as a host defense against siderophilic bacteria because of its ability to sequester iron-containing bacterial siderophores. LCN2 was also described as a bone-derived hormone, which crosses the BBB and suppresses appetite in a MC4R-dependent manner [7]. Here, we show that cranial infusion of LCN2 in WT mice was sufficient to induce some of the main molecular characteristics of cachexia such as endothelial inflammation, microglia activation and upregulation of oligodendrocyte specific genes, such as *Plin4*. In addition, we observed a robust activation of various components of the immune system, such as increased interleukin signaling, an IFG response and neutrophil degranulation, which is consistent with the described role of LCN2 as a neutrophil chemoattractant [32]. In accordance with this, TB Lcn2 KO mice showed a reduced induction of cachexia markers in endothelium, microglia, and oligodendrocytes during cachexia compared with TB WT mice. Interestingly, we could, for the first time, describe which genes are affected in food-intake regulating neurons in the hypothalamus by LCN2. Indeed, recent studies describe LCN2 as a bone-derived hormone suppressing food intake and it was proposed that this occurred via direct binding of LCN2 to the type 4 melanocortin receptor [7]. However, the observed binding of radiolabeled LCN2 to the hypothalamus may represent a combined contribution of more than one receptor type [9]. The data presented here suggest that LCN2 also acts directly on appetite-regulating neurons to exert another level of control on appetite during pathological states. Altogether, our LCN2 modulation studies give a precise picture of the role of LCN2 in the MBH and suggest that this protein could serve as a therapeutic target to abolish certain features of cachexia.

The induction of stress genes like *Lrrc8a*, *Mt1*, and *Cirbp* shows that various types of stress are induced in the MBH during cancer. *Lrrc8a* subunits are essential for the formation of volume-regulated anion channels, which regulate cell size and influx of small molecules. *Lrrc8a* expression is highly sensitive to oxidative stress [33], and its induction causes cell shrinkage mediated by an efflux of KCl and other osmolytes from cells [34]. Although such mechanisms most likely are meant to protect neurons from bursting, the effects can also be lethal for cells. The less optimal microenvironment for neurons under cachectic stress is also indicated by the induction of *Mt1* and *Mt2*, which function to neutralize free radicals generated by cellular stress, to aid in maintaining intracellular homeostasis [35]. *Cirbp* is upregulated under a variety of stress conditions, such as hypoxia and glucose deprivation, and may aid in coping with changes in cell health by triggering alternative splicing. Interestingly, *Cirbp* was reported to be upregulated upon food deprivation in Glu5 neurons [18], an effect proposed to be triggered by relative glucose depletion, and ultimately leading to the activation of orexigenic neurons in the hypothalamus.

Of interest is the induction of an inflammatory network in endothelial cells in the cachectic MBH, highlighted by ICAM-1^+^ blood vessels. With the induction of *Lcn2*, *Ly6a*, *Ly6c1*, *Vcam* and *Icam1*, the MBH micro-environment becomes a magnet for immune cells and indeed we previously reported the influx of immune cells in this region of the brain [27]. However, in our model, we did not detect such influx, likely because our sample represents a state of early cachexia with only minor inflammation, and because the total number of short-lived, dynamically infiltrating cells is low at any given time point relative to the other cells in this region.

Recently, several studies have analyzed microglia on the single cell level, greatly increasing our knowledge about this complex cell type [24,36]. It is now established that microglia actively contribute to brain maintenance as well as neurodegenerative diseases, and have different phenotypes throughout lifespan or in disease. Although most of the recent studies were performed on whole brain samples followed by Facs sorting and were not focused on the MBH, unique microglial stages were previously identified that resemble those found in our data. For example, a major finding in the study of Hammond et al. [36] was the discovery of an uncommon microglia subtype selectively expressing the chemokine CCL4 and containing many neurotoxic signals, such as TNF and ILB. This subtype is uniquely suited to produce an inflammatory response, attract peripheral immune cells into the CNS, and cause further brain damage. The IBA1^+^/CD45^+^ microglial subtype identified in our study resembles this “neurotoxic” pro-inflammatory microglial population, which contrasts with the likely function of IBA1^+^/CD45^−^ microglia that seem to support growth, development and survival of neurons. Recently, our group demonstrated that removal of all microglia by delivery of CSF1R inhibitors worsens the outcome of cachexia and we concluded that microglia are therefore neuroprotective [27], although the mechanism remained elusive. With our scRNA-seq we can now conclude that a neuroprotective microglia population in the MBH exists and may provide a local source of neurotrophic factors and anti-inflammatory signals that are released under cachectic stress. We identified three separate induced gene networks that are predicted to be involved in neuronal support, cell signaling or ECM organization. Indeed, previous reports showed that microglia can release neurotrophic factors to facilitate neuronal functioning [37,38]. Blocking of the IBA1^+^/CD45^+^ microglia in cachexia, while leaving the IBA1^+^/CD45^−^ unaffected, could be of interest to further improve the outcome in this condition.

Historically, oligodendrocytes were solely assigned a role in the production of myelin, but new insights indicate that oligodendrocytes produce neurotrophic factors, mediate leptin sensing, and are involved in the regulation of energy homeostasis by providing lactate and pyruvate produced during glycolysis [39]. These metabolites in turn influence food intake by regulating neurons leading to a decreased appetite. In addition, a recent study showed that refeeding after an overnight fast rapidly triggers proliferation and differentiation of oligodendrocyte progenitors in the MBH [14]. In accordance with this, our data showed that oligodendrocytes under cachectic stress activate genes involved in lipid catabolism and we hypothesize that this ultimately leads to the induction of *Plin4*, a novel biomarker for oligodendrocytes under cachectic stress. PLIN4 induction is required for deposition of intracellular lipid droplets and may precede lipotoxicity and neurodegenerative disease. Indeed, PLIN4 is upregulated in a mouse model of multiple sclerosis [40] and Parkinson's disease where it appears to impact tyrosine-hydroxylase (TH)^+^ dopaminergic neuronal function [41]. It is possible that such interactions between lipid droplets and TH^+^ neurons also take place in orexigenic TH positive neurons in the MBH, ultimately leading to decreased food intake [42]. More evidence for enhanced signaling between oligodendrocytes and other cell types during cachectic stress is provided by the induction of *Ptgds*. PTGDS is an enzyme which catalyzes the conversion of prostaglandin H2 to the neuromodulator or neuroinflammatory molecule prostaglandin D2 that is in turn known to cause demyelination and astrogliosis, which are commonly observed in neurodegenerative diseases [43].

Altogether, our data show that the MBH is a critical regulator of cancer cachexia, orchestrated by molecular changes in multiple cell types. Although a great physical distance exists between the MBH and the peripheral tumor, many features observed in the TME can be found in the MBH as well. Several potential mediators were identified in pancreatic cancer, such as LRG1 [44] and LCN2 [45], which can cross the attenuated BBB in the MBH. Another potential mediator of cachectic stress maybe CCR1+ myeloid cells found in both the TME and the MBH carrying inflammatory molecules like TNF, IL1B, and IL6. It is likely that these myeloid cells and blood borne biomarkers of pancreatic cancer act in symphony to induce some of the observed local changes in the MBH, which is confirmed by the high level of co-regulation between a number of these transcripts (e.g. *lrg1*, *Plin4*, *Lcn2* and *Icam1*). These factors have the potential as therapeutic targets to alleviate the symptoms of cachexia.

## 4 Methods

### 4.1 Animals: murine PDAC cachexia model

Our lab generated a mouse model of PDAC-associated cachexia by the single orthotopic (OT) implantation of murine-derived C57BL/6 KRAS^G12D^ P53^R172H^ Pdx-Cre^+/+^ (KPC) pancreatic ductal adenocarcinoma (PDAC) [15]. These cells were derived from tumors in C57BL/6 mice heterozygous for oncogenic KRAS^G12D^ and point mutant TP53^R172H^ (both downstream of lox-stop-lox cassettes) with expression induced and targeted to the pancreas via the PDX-1-Cre driver [16]. All mice were maintained on a normal 12 h light, 12 h dark cycle with *ad libitum* access to normal chow and water. WT mice, Lcn2 KO mice, and *Myd88* KO mice were between 70 and 90 days old at study initiation. Mice received orthotopic implantation by injecting 1 million KPC cells in the tail of the pancreas adjacent to the lower end of the spleen, while mice were anesthetized with isoflurane. Sham mice underwent an identical surgical procedure, but the pancreas was injected with a similar volume of PBS. At a minimum of four days prior to implantation, mice were single housed and food intake was measured daily starting 24 h after implantation. WT, Lcn2 KO, and Myd88 KO mice were obtained from the Jackson Laboratories (Bar Harbor, ME, USA).

### 4.2 Intracerebroventricular injections of LCN2

Mice were anesthetized using isoflurane on a stereotactic alignment instrument (Kopf Instruments). Bregma was exposed with a 3 mm incision, and a 26-gauge lateral ventricle cannula was placed and affixed with acrylic at 1.0 mm X, −0.5 mm Y, and −2.25 mm Z relative to bregma. Mice recovered for one week after cannulation surgery, then recombinant mouse LCN2 (R&D Systems, 40 ng) was diluted in artificial CSF and injected in a total volume of 2 uL for 10 consecutive days. LPS treatment was done by intraperitoneal injection (2 mg/kg, *Escherichia coli*, 055:B5, Sigma), mice were euthanized 24 h later, and the hypothalamus was used for analysis.

### 4.3 Cell culture

Cells were maintained in RPMI (Gibco) supplemented with 10% heat-inactivated FBS, and 50 U/mL penicillin/streptomycin (Gibco, Thermo Fisher), in incubators maintained at 37 °C and 5% CO_2_.

### 4.4 Single-cell RNA-seq library generation of the MBH

Sham and TB mice were euthanized 20 days after the implantation of KPC-implantation (Figure 1A). Precise dissection of the arcuate nucleus and surrounding structures was achieved using a chilled steel brain matrix (cat: SA-2165, Roboz Surgical Instrument Co., Gaithersburg, MD) and a surgical-grade knives molded to a 90° angle. Accurate dissection of the arcuate nucleus was confirmed by utilizing POMC-eGFP mice (Jax-009593) (Figure S1A). Hypothalami from sham and TB mice were then pooled separately, dissociated using Papain digestion, and triturated using fire polished Pasteur pipettes tapered to sequentially decreasing-sized openings. The resulting single cell suspension was used to generate two 3′-end scRNA-seq 10X Genomics libraries, each containing ∼6,000 cells. The libraries were then sequenced with NextSeq HighOutput PE150 with 400 million reads per sample as we did previously [11].

### 4.5 Single-cell RNA-seq data processing

Sequence reads were aligned with Cell Ranger v3 to create a gene expression count matrix, and Seurat 3.2.2. [46] was used for further downstream analysis. Poor quality cells, indicated by high mitochondrial content (>15%) or low number of genes (<300), were removed, resulting in a total of 3854 cells for sham and 3282 cells for the TB mice. Data from sham and TB mice were then normalized using the function normalizeData, and variable features of both sham and TB mice samples were identified with the VST method (nfeatures = 2000). To combine the samples to obtain similar clustering, we identified integration anchors using the function FindIntegrationAnchors and then integrated the data using the function IntegrateData. The combined sham-TB mice matrix was then scaled, and clustering was obtained using the standard Seurat workflow via the functions RunPCA, RunTSNE (35 PCs), FindNeighbors (15 dims) and FindClusters (resolution = 0.6). With these settings, the cells were assigned into 20 different clusters, with each cluster containing cells from both the sham and TB mice group. Classification of each cluster was then performed by finding marker genes for every cluster with the FindMarker function and cross-referenced with known cell marker genes based on existing literature. Most of the clusters contained one or more specific markers, indicating that those clusters can be considered a separate cell type. Differences between sham and TB mice were found using the function FindMarkers and standard statistical testing was employed using the Wilcoxon rank sum test. Cell–cell communication mediated by ligand–receptor complexes was established with the Python package CellPhoneDB as described previously [28]. First, mouse genes were converted to their human orthologs, and those without human orthologs were excluded from analysis. For interpretation and visualization of DE genes, gene networks were generated using unbiased clustering based on co-expresssion data of 31,499 human public RNA-seq samples [47]. Mouse genes, which have no human orthologs, were excluded. All networks were based on the co-expression of human genes.

### 4.6 RNA isolation and quantitative RT-PCR

Total RNA was isolated with the RNeasy mini kit (Qiagen) according to manufacturer's directions and was converted to cDNA with the Taqman cDNA synthesis kit. 20 ng cDNA was used for qPCR with the Roche 96 light cycler. Primers for amplification of genes are listed in Supplementary Table 1. mRNA expression values were normalized to *GAPDH* using the ΔΔCt method. Fold inductions and relative expression levels were calculated from three or more individual experiments.

### 4.7 RNA sequencing

Hypothalami were isolated after intracerebroventricular injections of LCN2 or vehicle, and RNA was isolated. Library preparation and sequencing were performed by BGI Genomics at 100 base paired-end sequencing with 20M clean reads per sample. HISAT was used to align the clean reads to the reference genome (GRCm38), with an average mapping ratio of 96.53%. Bowtie2 was used to align the reads to reference genes, resulting in the detection of 18336 genes. A count matrix was then generated and imported into DESeq2 for differential analyses with default settings. After generating a result table, we constructed a co-expression network using the top-induced genes. Heatmaps were generated using the most variable genes, as well as using genes involved in relevant pathways. The result table was then imported in the package EnhancedVolcano to generate a Volcano plot.

### 4.8 Immunohistochemistry (IHC)

Mice were intraperitoneally injected with a ketamine cocktail before performing perfusion with PBS and 4% PFA. Brains were then fixed in 4% PFA overnight, cryoprotected with sucrose gradients (5%, 10%, 15%, and 30% sucrose), and frozen in OCT blocks, followed by sectioning with a cryostat (15 μm sections) or a microtome (30 μm sections). IHC for the 15 μm sections was performed by incubating brain sections with commercial antibodies for IBA1 (Wako, NCNP24), NPY (Peninsula Laboratories, T-4070), POMC (Phoenix Pharmaceuticals) or LCN2 (R&D, MAB1857) and VWF (Abcam, ab11713) in blocking buffer (PBS + 0.1% Triton X-100 + 2% BSA + 0.02% Tween20) overnight at 4 °C. The next day, slices were washed with PBT and incubated with secondary fluorescence antibodies in blocking buffer followed by washing and counter staining with DAPI. IHC for the 30 μm sections was performed by incubating them with commercial antibodies for CD45 (BD, rat, 30-F11) and IBA1 (Wako, NCNP24) or PECAM1 (BD, 550274) and ICAM-1 (R&D, AF796) in blocking buffer (PBS + 5% donkey serum + 0.3% Triton X-100) overnight at 4 °C. Next day, slices were washed with PBT and incubated with secondary fluorescence antibodies in blocking buffer followed by washing and counter staining with DAPI. Microscope images were taken using a Nikon confocal microscope.

### 4.9 ISH using dig-labeled probes

Mice were intraperitoneally injected with a ketamine cocktail before performing perfusion with PBS and 4% PFA. ISH was then performed as previously described [48]. Brains were fixed in PBS + 4% PFA overnight, cryoprotected with sucrose gradients (5%, 10%, 15%, and 30% sucrose), and frozen in OCT blocks, followed by sectioning with cryostat (15 μm per section), and subsequently frozen again. ISH was then performed by defrosting the sections at RT, followed by incubation in PBS + 4% PFA for 10 min, washing in PBS for 3 × 5 min, incubation in Proteinase K buffer for 10 min, incubation in PBS + 4% PFA for 5 min, washing in PBS for 3 × 5 min, incubation in acetylation buffer for 10 min, incubation in hybridization buffer for 1 h followed by hybridization at 68 °C overnight with indicated RNA probes dissolved in hybridization buffer (100 μl per slide and covered with cover glass to prevent drying out). After hybridization, slices were incubated in in MAPT buffer for 10 min and cover slips were removed. Then, slices were washed in washing buffer (50% formamide, 1× SSC solution and 0.1% Tween20) for 1 h at 65 °C, blocked in MABT buffer + 4% BSA for 1 h and incubated with an anti-digoxigenin-AP antibody (11093274910 Roche, 1:5000) in MABT buffer + 2% BSA overnight at 4 °C. Next day, cells were washed with MABT buffer for 3 × 5 min and color reaction was performed in developing buffer (100 mM Tris pH9.8, 100 mM NaCl, 50 mM MgCl2) supplemented with NBT/BCIP for 1–3 days. Reaction was stopped by adding PBS. Pictures were subsequently taken with a Leica DM4 B microscope.

For quantification of *Aldoc* and *Mt1*, microscopic images were converted in black and white and subsequently inverted. Then, the intregrated density was measured using Image J software of similar regions of the MBH between sham and TB mice (n = 3). For the quantification of *Pgtds*, the positive cells in the MBH were counted (n = 3).

### 4.10 ISH using radioactively labeled probes

Brains were removed and snap frozen and stored at −80 °C. Coronal brainstem sections (20 μm) were cut on a cryostat and thaw-mounted onto Superfrost Plus slides (VWR Scientific, West Chester, PA). Antisense 33S-labeled Plin4 (concentration 0.1 pmol/mL) was denatured, dissolved in hybridization buffer along with tRNA (1.7 mg/mL), and applied to slides. Slides were covered with glass coverslips, placed in a humid chamber, and incubated overnight at 55 °C. The following day, slides were treated with RNase A and washed under conditions of increasing stringency. Slides were dipped in 100% ethanol, air dried, and then dipped in NTB liquid emulsion (Carestream Health, Inc., Rochester, NY). Slides were developed 5–7 days later and coverslipped. Silver grains were viewed with a Carl Zeiss Axioskop microscope (Carl Zeiss, Thornwood, NY) and representative pictures were taken using a Cohu 4910 camera (San Diego, CA).

ISH probes were generated by converting hypothalamic RNA of TB mice to cDNA and subsequent PCR using the primers in Supplementary Table 1. PCR products were then digested with the indicated enzymes and ligated into the PDP18 plasmid (from Ambion). RNA probes were then generated using T7 RNA polymerase followed by column purification; for radioactively labeled probes: NucAway Spin Columns (Initrogen, AM10070), for dig-labeled probes: MEGAclear Kit (Initrogen, AM1908).

### 4.11 Quantification and statistical analysis

Significance p values are indicated in the text and figure legends. Error bars in the experiments represent standard error of the mean (SEM). Student's t test was used for comparisons between conditions. Statistical analyses were performed using GraphPad Prism 5.0.

## Author contributions

Christian measured food intake, prepared samples for scRNA-seq, performed the bioinformatics analysis, the ISH with riboprobes, IHC, qPCR, and wrote the manuscript. Brannon did the orthotopic implantation of KPC cells, assisted in preparing the scRNA-seq samples, performed the LCN2 KO mice studies, performed intracerebroventricular injections of LCN2, and edited the manuscript. Mason assisted with the IHC, and Peter performed ISH with radioactively labeled probes. Monique assisted with the bioinformatics analysis and edited the manuscript. Stephanie edited the manuscript. Dan supervised the experiments, helped interpret the data, and edited the manuscript.

## Appendix A Supplementary data

Multimedia component 1 Supplementary Table 2: Cluster specific genes found with the scRNA-seq

Multimedia component 2 Supplementary Table 4: Conserved genes between sham and TB-mice per cluster

Multimedia component 3 Supplementary Table 5: Results table of the RNA-seq of the hypothalamus after LCN2 treatment

Multimedia component 4 Supplementary Table 6: Potential ligand–receptor interactions between the clusters

Multimedia component 5 Supplementary Table 1: Primers used in this study

Multimedia component 6 Supplementary Table 3: Bulk- and cell type-specific DE genes
