# Supplementary material for: Critical changes in hypothalamic gene networks in response to pancreatic cancer as found by single-cell RNA sequencing
Source: Mol Metab. 2022 Jan 11;58:101441. doi: 10.1016/j.molmet.2022.101441 (PMC8851272; doi:10.1016/j.molmet.2022.101441)
Supplement: Multimedia component 1 [file mmc1.pptx]

## Slide 1
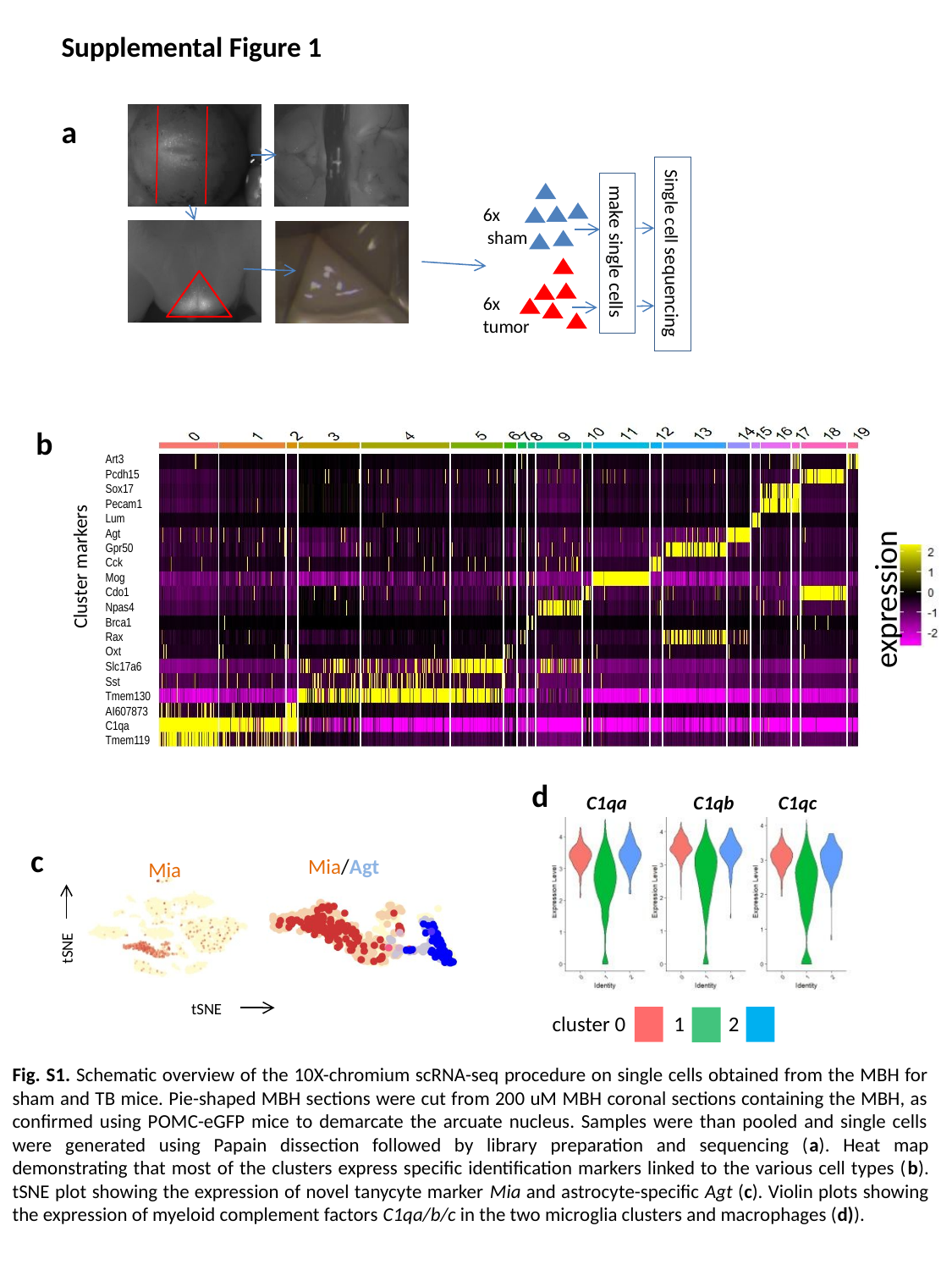

Supplemental Figure 1
a
6x
 sham
6x
tumor
make single cells
Single cell sequencing
b
Art3
Pcdh15
Sox17
Pecam1
Lum
Agt
Gpr50
Cck
Mog
Cdo1
Npas4
Brca1
Rax
Oxt
Slc17a6
Sst
Tmem130
AI607873
C1qa
Tmem119
Cluster markers
expression
d
C1qa C1qb C1qc
c
Mia/Agt
Mia
tSNE
tSNE
cluster 0 1 2
Fig. S1. Schematic overview of the 10X-chromium scRNA-seq procedure on single cells obtained from the MBH for sham and TB mice. Pie-shaped MBH sections were cut from 200 uM MBH coronal sections containing the MBH, as confirmed using POMC-eGFP mice to demarcate the arcuate nucleus. Samples were than pooled and single cells were generated using Papain dissection followed by library preparation and sequencing (a). Heat map demonstrating that most of the clusters express specific identification markers linked to the various cell types (b). tSNE plot showing the expression of novel tanycyte marker Mia and astrocyte-specific Agt (c). Violin plots showing the expression of myeloid complement factors C1qa/b/c in the two microglia clusters and macrophages (d)).

## Slide 2
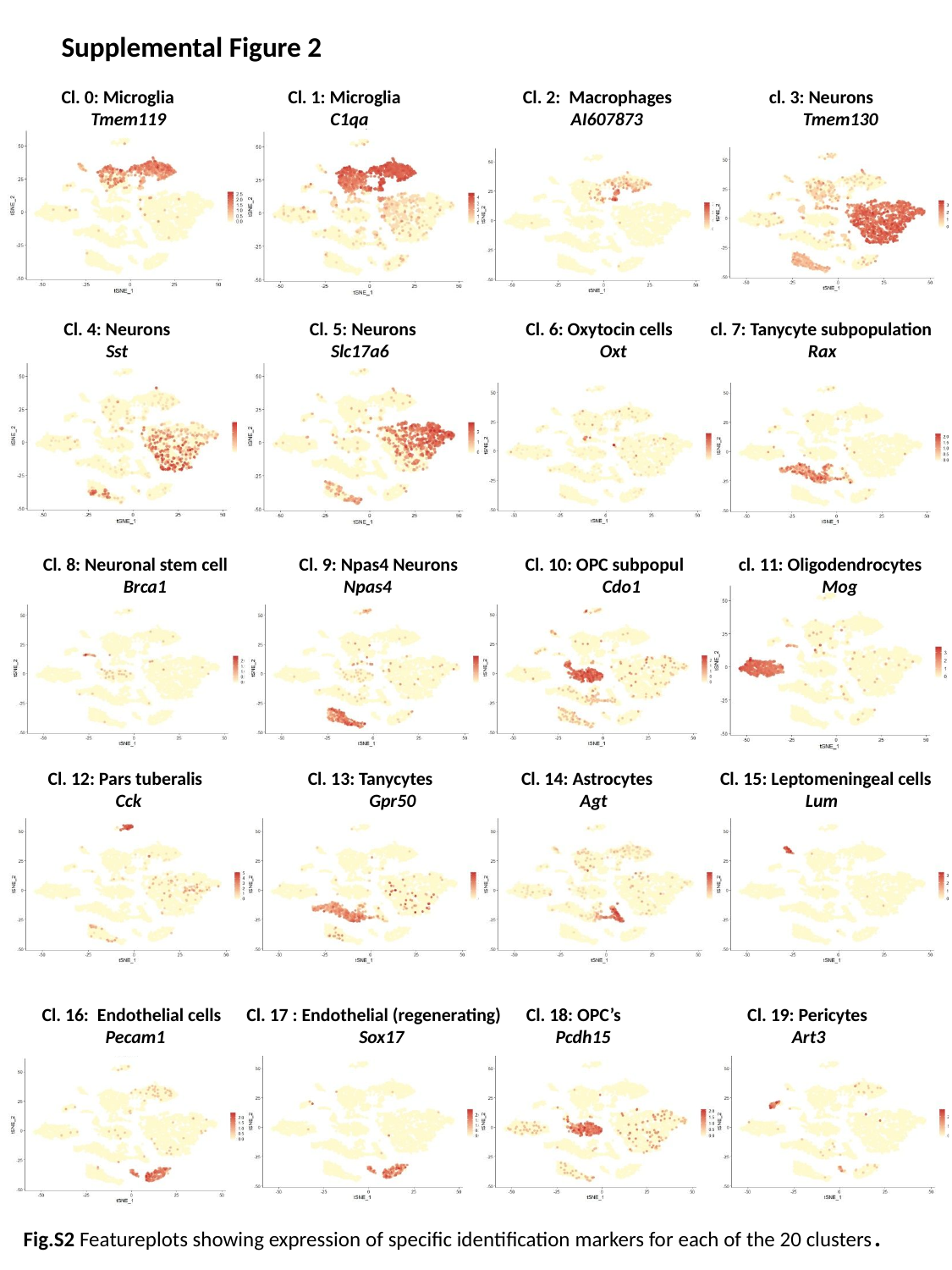

Supplemental Figure 2
Cl. 0: Microglia Cl. 1: Microglia Cl. 2: Macrophages cl. 3: Neurons
 Tmem119 C1qa AI607873 Tmem130
Cl. 4: Neurons Cl. 5: Neurons Cl. 6: Oxytocin cells cl. 7: Tanycyte subpopulation
 Sst Slc17a6 Oxt Rax
Cl. 8: Neuronal stem cell Cl. 9: Npas4 Neurons Cl. 10: OPC subpopul cl. 11: Oligodendrocytes
 Brca1 Npas4 Cdo1 Mog
Cl. 12: Pars tuberalis Cl. 13: Tanycytes Cl. 14: Astrocytes Cl. 15: Leptomeningeal cells
 Cck Gpr50 Agt Lum
Cl. 16: Endothelial cells Cl. 17 : Endothelial (regenerating) Cl. 18: OPC’s Cl. 19: Pericytes
 Pecam1 Sox17 Pcdh15 Art3
Fig.S2 Featureplots showing expression of specific identification markers for each of the 20 clusters.

## Slide 3
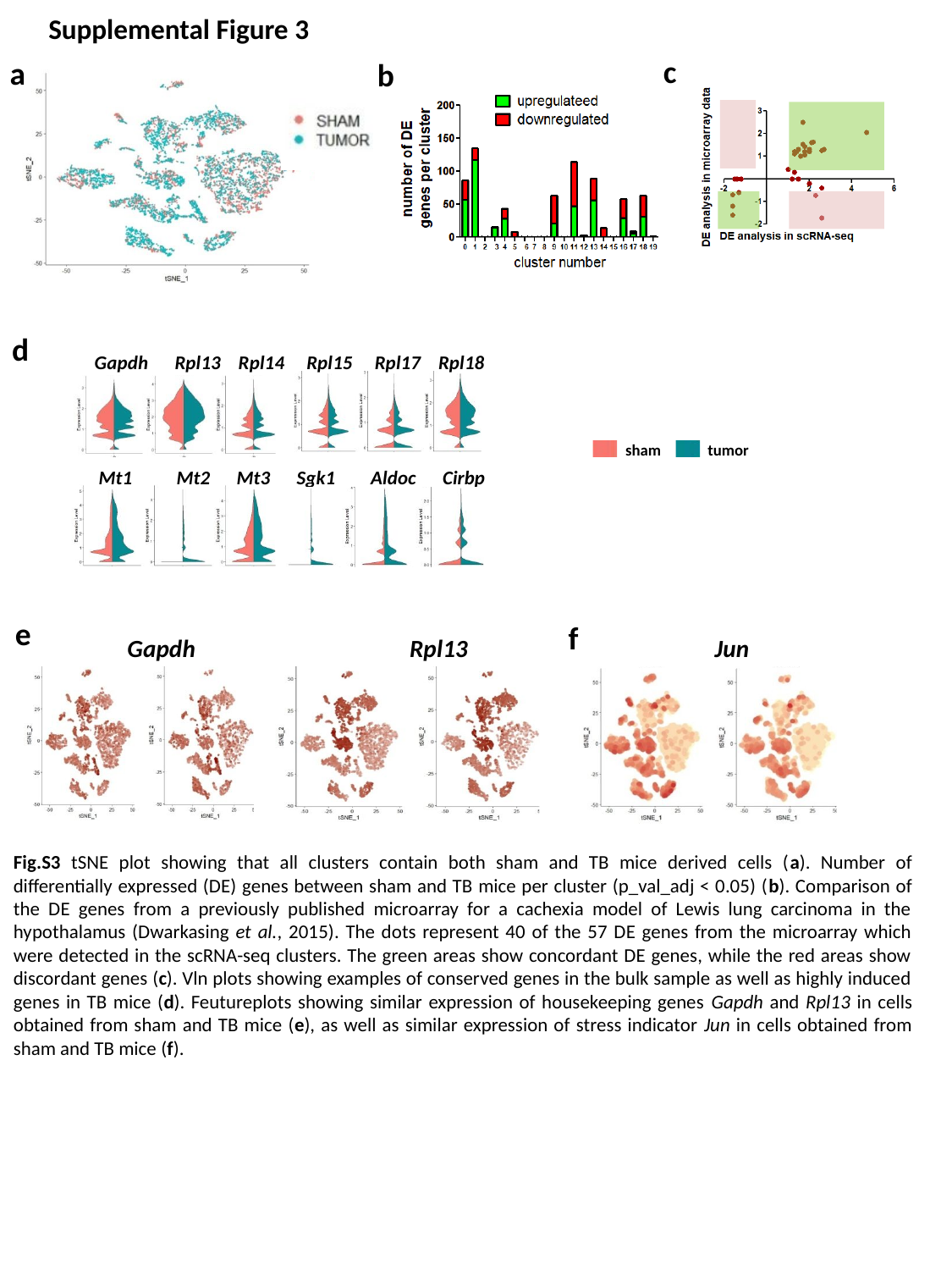

Supplemental Figure 3
c
a
b
d
Gapdh Rpl13 Rpl14 Rpl15 Rpl17 Rpl18
sham tumor
Mt1 Mt2 Mt3 Sgk1 Aldoc Cirbp
a
e
f
Gapdh Rpl13 Jun
Fig.S3 tSNE plot showing that all clusters contain both sham and TB mice derived cells (a). Number of differentially expressed (DE) genes between sham and TB mice per cluster (p_val_adj < 0.05) (b). Comparison of the DE genes from a previously published microarray for a cachexia model of Lewis lung carcinoma in the hypothalamus (Dwarkasing et al., 2015). The dots represent 40 of the 57 DE genes from the microarray which were detected in the scRNA-seq clusters. The green areas show concordant DE genes, while the red areas show discordant genes (c). Vln plots showing examples of conserved genes in the bulk sample as well as highly induced genes in TB mice (d). Feutureplots showing similar expression of housekeeping genes Gapdh and Rpl13 in cells obtained from sham and TB mice (e), as well as similar expression of stress indicator Jun in cells obtained from sham and TB mice (f).

## Slide 4
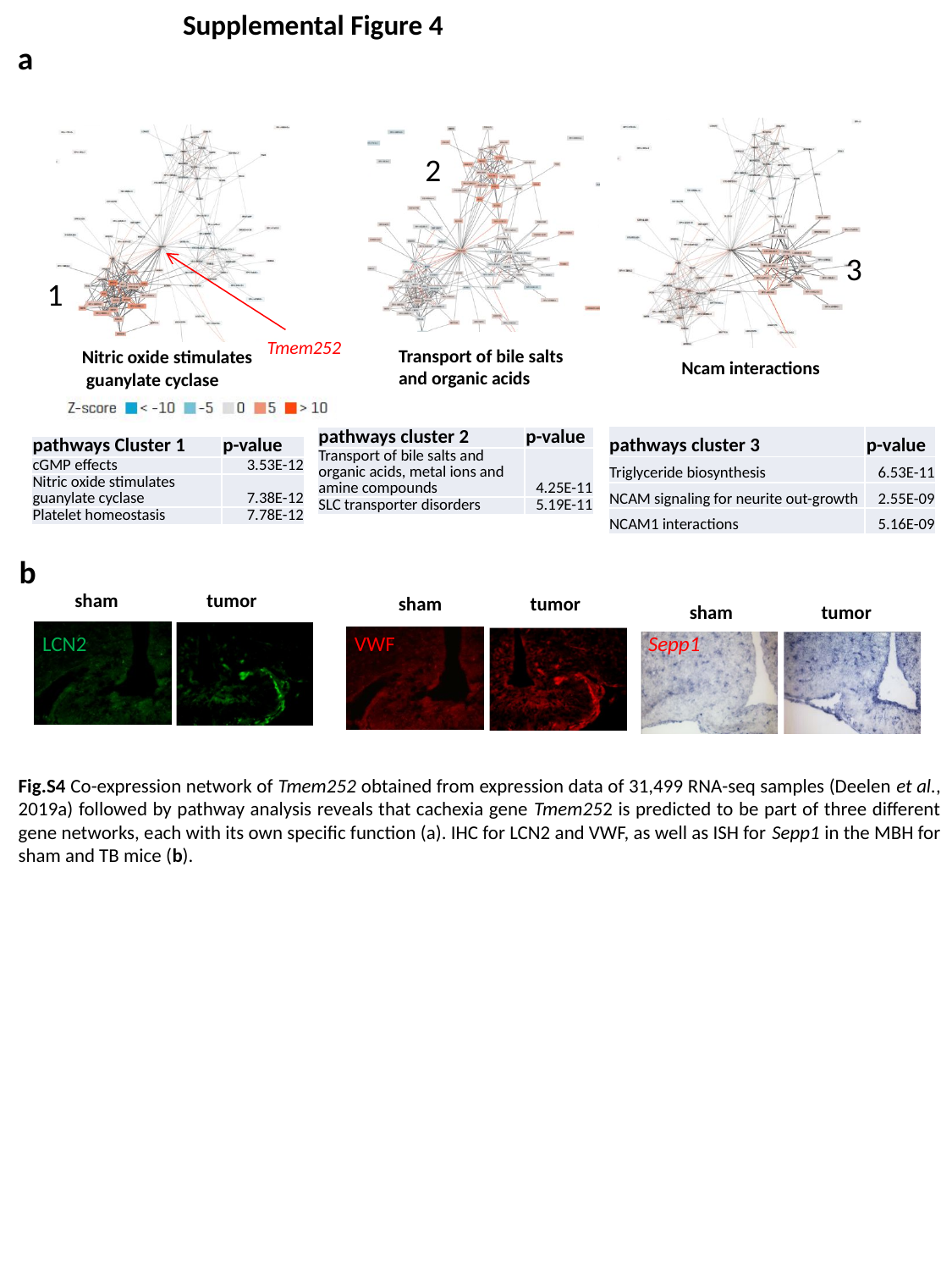

Supplemental Figure 4
a
2
3
1
Tmem252
Transport of bile salts
and organic acids
Nitric oxide stimulates
 guanylate cyclase
Ncam interactions
| pathways cluster 3 | p-value |
| --- | --- |
| Triglyceride biosynthesis | 6.53E-11 |
| NCAM signaling for neurite out-growth | 2.55E-09 |
| NCAM1 interactions | 5.16E-09 |
| pathways cluster 2 | p-value |
| --- | --- |
| Transport of bile salts and organic acids, metal ions and amine compounds | 4.25E-11 |
| SLC transporter disorders | 5.19E-11 |
| pathways Cluster 1 | p-value |
| --- | --- |
| cGMP effects | 3.53E-12 |
| Nitric oxide stimulates guanylate cyclase | 7.38E-12 |
| Platelet homeostasis | 7.78E-12 |
b
sham tumor
sham tumor
sham tumor
LCN2 VWF Sepp1
Fig.S4 Co-expression network of Tmem252 obtained from expression data of 31,499 RNA-seq samples (Deelen et al., 2019a) followed by pathway analysis reveals that cachexia gene Tmem252 is predicted to be part of three different gene networks, each with its own specific function (a). IHC for LCN2 and VWF, as well as ISH for Sepp1 in the MBH for sham and TB mice (b).

## Slide 5
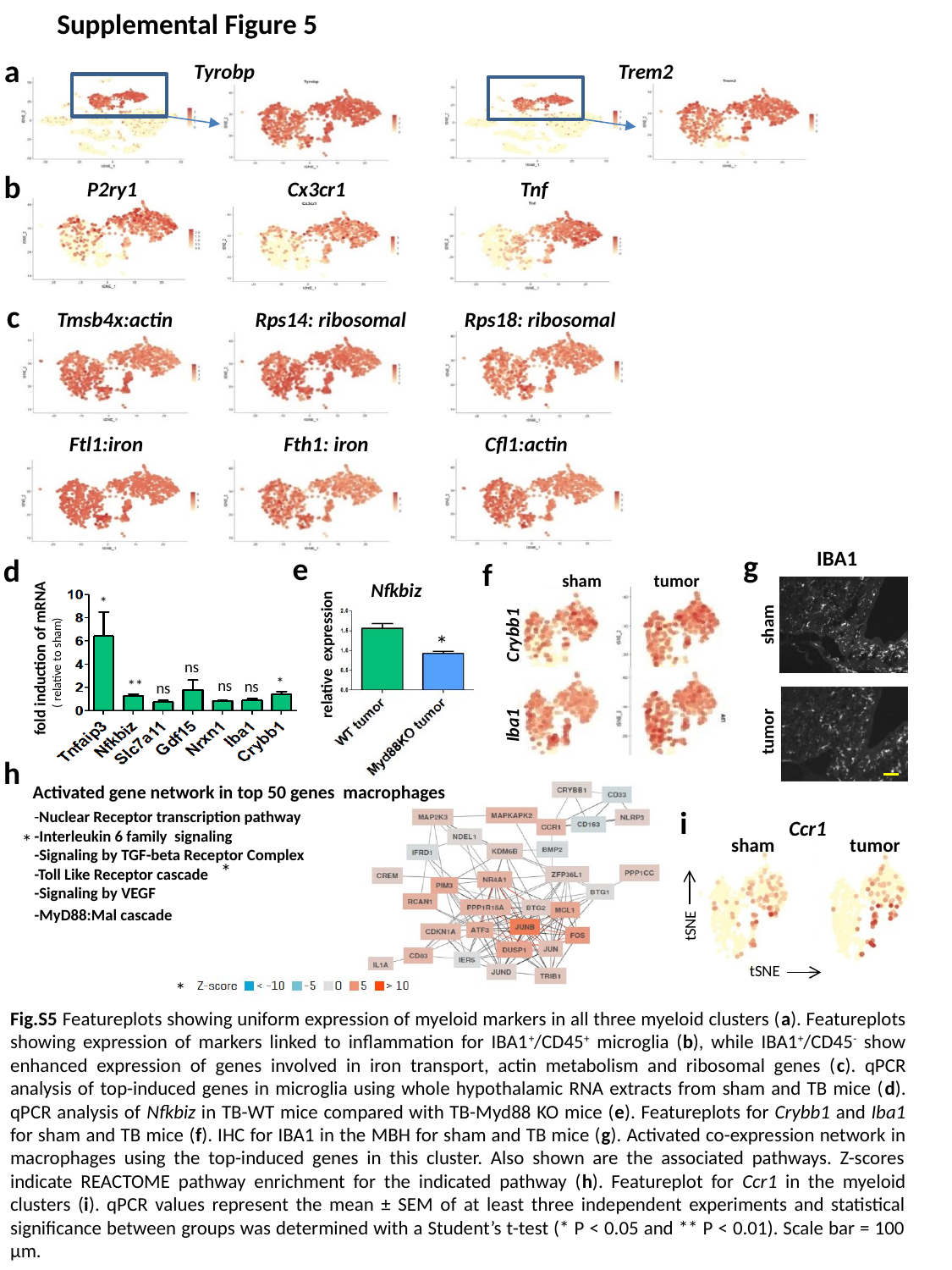

Supplemental Figure 5
a
Tyrobp Trem2
b
 P2ry1 Cx3cr1 Tnf
c
Tmsb4x:actin Rps14: ribosomal Rps18: ribosomal
Ftl1:iron Fth1: iron Cfl1:actin
IBA1
g
e
d
f
sham tumor
Nfkbiz
*
fold induction of mRNA
 ( relative to sham)
relative expression
tumor sham
*
Iba1 Crybb1
ns
*
**
ns
ns
ns
h
Activated gene network in top 50 genes macrophages
i
-Nuclear Receptor transcription pathway
-Interleukin 6 family signaling
-Signaling by TGF-beta Receptor Complex
-Toll Like Receptor cascade
-Signaling by VEGF
-MyD88:Mal cascade
Ccr1
*
sham tumor
*
tSNE
tSNE
*
Fig.S5 Featureplots showing uniform expression of myeloid markers in all three myeloid clusters (a). Featureplots showing expression of markers linked to inflammation for IBA1+/CD45+ microglia (b), while IBA1+/CD45- show enhanced expression of genes involved in iron transport, actin metabolism and ribosomal genes (c). qPCR analysis of top-induced genes in microglia using whole hypothalamic RNA extracts from sham and TB mice (d). qPCR analysis of Nfkbiz in TB-WT mice compared with TB-Myd88 KO mice (e). Featureplots for Crybb1 and Iba1 for sham and TB mice (f). IHC for IBA1 in the MBH for sham and TB mice (g). Activated co-expression network in macrophages using the top-induced genes in this cluster. Also shown are the associated pathways. Z-scores indicate REACTOME pathway enrichment for the indicated pathway (h). Featureplot for Ccr1 in the myeloid clusters (i). qPCR values represent the mean ± SEM of at least three independent experiments and statistical significance between groups was determined with a Student’s t-test (* P < 0.05 and ** P < 0.01). Scale bar = 100 μm.

## Slide 6
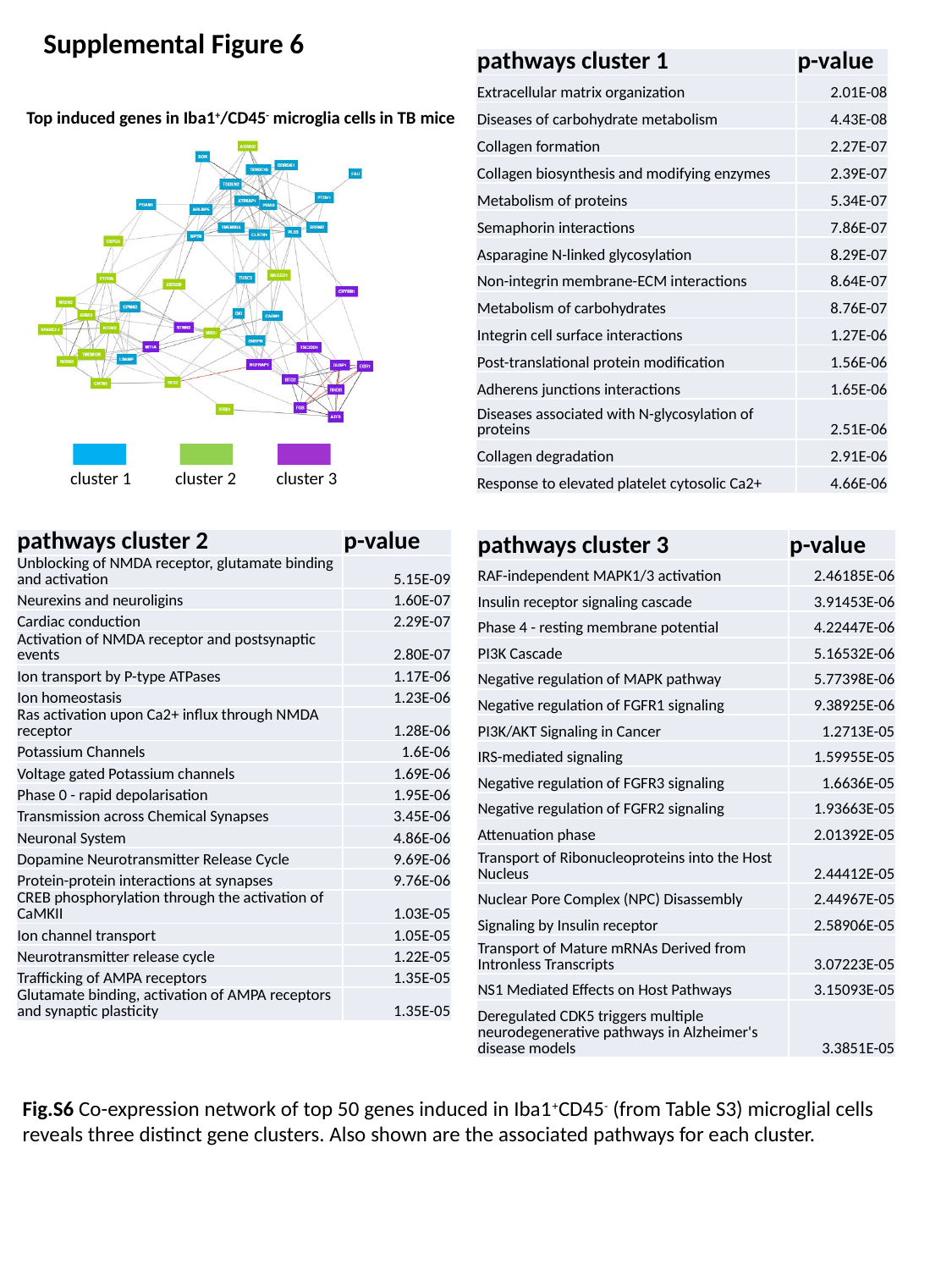

Supplemental Figure 6
| pathways cluster 1 | p-value |
| --- | --- |
| Extracellular matrix organization | 2.01E-08 |
| Diseases of carbohydrate metabolism | 4.43E-08 |
| Collagen formation | 2.27E-07 |
| Collagen biosynthesis and modifying enzymes | 2.39E-07 |
| Metabolism of proteins | 5.34E-07 |
| Semaphorin interactions | 7.86E-07 |
| Asparagine N-linked glycosylation | 8.29E-07 |
| Non-integrin membrane-ECM interactions | 8.64E-07 |
| Metabolism of carbohydrates | 8.76E-07 |
| Integrin cell surface interactions | 1.27E-06 |
| Post-translational protein modification | 1.56E-06 |
| Adherens junctions interactions | 1.65E-06 |
| Diseases associated with N-glycosylation of proteins | 2.51E-06 |
| Collagen degradation | 2.91E-06 |
| Response to elevated platelet cytosolic Ca2+ | 4.66E-06 |
Top induced genes in Iba1+/CD45- microglia cells in TB mice
cluster 1 cluster 2 cluster 3
| pathways cluster 2 | p-value |
| --- | --- |
| Unblocking of NMDA receptor, glutamate binding and activation | 5.15E-09 |
| Neurexins and neuroligins | 1.60E-07 |
| Cardiac conduction | 2.29E-07 |
| Activation of NMDA receptor and postsynaptic events | 2.80E-07 |
| Ion transport by P-type ATPases | 1.17E-06 |
| Ion homeostasis | 1.23E-06 |
| Ras activation upon Ca2+ influx through NMDA receptor | 1.28E-06 |
| Potassium Channels | 1.6E-06 |
| Voltage gated Potassium channels | 1.69E-06 |
| Phase 0 - rapid depolarisation | 1.95E-06 |
| Transmission across Chemical Synapses | 3.45E-06 |
| Neuronal System | 4.86E-06 |
| Dopamine Neurotransmitter Release Cycle | 9.69E-06 |
| Protein-protein interactions at synapses | 9.76E-06 |
| CREB phosphorylation through the activation of CaMKII | 1.03E-05 |
| Ion channel transport | 1.05E-05 |
| Neurotransmitter release cycle | 1.22E-05 |
| Trafficking of AMPA receptors | 1.35E-05 |
| Glutamate binding, activation of AMPA receptors and synaptic plasticity | 1.35E-05 |
| pathways cluster 3 | p-value |
| --- | --- |
| RAF-independent MAPK1/3 activation | 2.46185E-06 |
| Insulin receptor signaling cascade | 3.91453E-06 |
| Phase 4 - resting membrane potential | 4.22447E-06 |
| PI3K Cascade | 5.16532E-06 |
| Negative regulation of MAPK pathway | 5.77398E-06 |
| Negative regulation of FGFR1 signaling | 9.38925E-06 |
| PI3K/AKT Signaling in Cancer | 1.2713E-05 |
| IRS-mediated signaling | 1.59955E-05 |
| Negative regulation of FGFR3 signaling | 1.6636E-05 |
| Negative regulation of FGFR2 signaling | 1.93663E-05 |
| Attenuation phase | 2.01392E-05 |
| Transport of Ribonucleoproteins into the Host Nucleus | 2.44412E-05 |
| Nuclear Pore Complex (NPC) Disassembly | 2.44967E-05 |
| Signaling by Insulin receptor | 2.58906E-05 |
| Transport of Mature mRNAs Derived from Intronless Transcripts | 3.07223E-05 |
| NS1 Mediated Effects on Host Pathways | 3.15093E-05 |
| Deregulated CDK5 triggers multiple neurodegenerative pathways in Alzheimer's disease models | 3.3851E-05 |
Fig.S6 Co-expression network of top 50 genes induced in Iba1+CD45- (from Table S3) microglial cells reveals three distinct gene clusters. Also shown are the associated pathways for each cluster.

## Slide 7
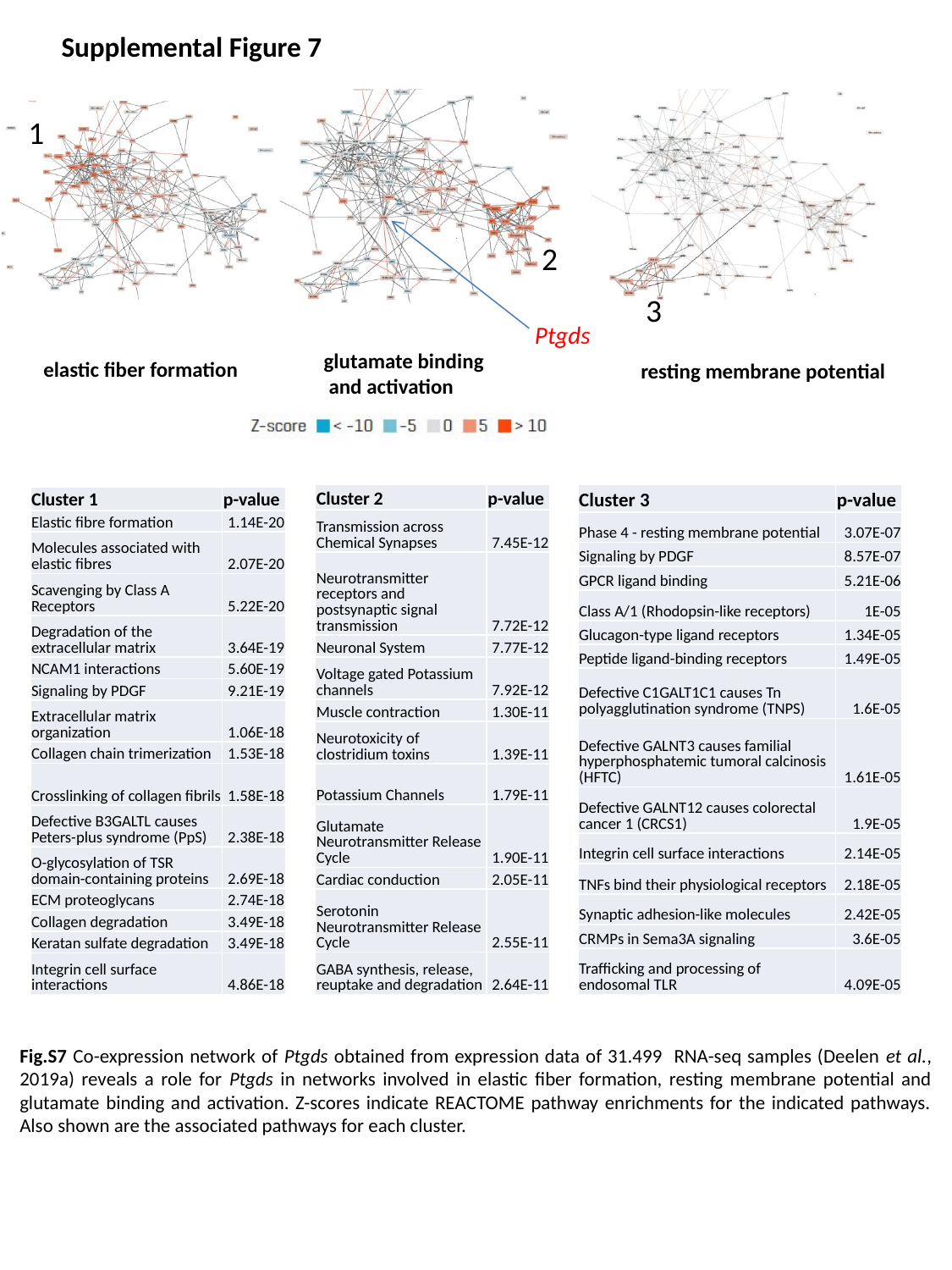

Supplemental Figure 7
1
2
3
Ptgds
glutamate binding
 and activation
elastic fiber formation
resting membrane potential
| Cluster 2 | p-value |
| --- | --- |
| Transmission across Chemical Synapses | 7.45E-12 |
| Neurotransmitter receptors and postsynaptic signal transmission | 7.72E-12 |
| Neuronal System | 7.77E-12 |
| Voltage gated Potassium channels | 7.92E-12 |
| Muscle contraction | 1.30E-11 |
| Neurotoxicity of clostridium toxins | 1.39E-11 |
| Potassium Channels | 1.79E-11 |
| Glutamate Neurotransmitter Release Cycle | 1.90E-11 |
| Cardiac conduction | 2.05E-11 |
| Serotonin Neurotransmitter Release Cycle | 2.55E-11 |
| GABA synthesis, release, reuptake and degradation | 2.64E-11 |
| Cluster 3 | p-value |
| --- | --- |
| Phase 4 - resting membrane potential | 3.07E-07 |
| Signaling by PDGF | 8.57E-07 |
| GPCR ligand binding | 5.21E-06 |
| Class A/1 (Rhodopsin-like receptors) | 1E-05 |
| Glucagon-type ligand receptors | 1.34E-05 |
| Peptide ligand-binding receptors | 1.49E-05 |
| Defective C1GALT1C1 causes Tn polyagglutination syndrome (TNPS) | 1.6E-05 |
| Defective GALNT3 causes familial hyperphosphatemic tumoral calcinosis (HFTC) | 1.61E-05 |
| Defective GALNT12 causes colorectal cancer 1 (CRCS1) | 1.9E-05 |
| Integrin cell surface interactions | 2.14E-05 |
| TNFs bind their physiological receptors | 2.18E-05 |
| Synaptic adhesion-like molecules | 2.42E-05 |
| CRMPs in Sema3A signaling | 3.6E-05 |
| Trafficking and processing of endosomal TLR | 4.09E-05 |
| Cluster 1 | p-value |
| --- | --- |
| Elastic fibre formation | 1.14E-20 |
| Molecules associated with elastic fibres | 2.07E-20 |
| Scavenging by Class A Receptors | 5.22E-20 |
| Degradation of the extracellular matrix | 3.64E-19 |
| NCAM1 interactions | 5.60E-19 |
| Signaling by PDGF | 9.21E-19 |
| Extracellular matrix organization | 1.06E-18 |
| Collagen chain trimerization | 1.53E-18 |
| Crosslinking of collagen fibrils | 1.58E-18 |
| Defective B3GALTL causes Peters-plus syndrome (PpS) | 2.38E-18 |
| O-glycosylation of TSR domain-containing proteins | 2.69E-18 |
| ECM proteoglycans | 2.74E-18 |
| Collagen degradation | 3.49E-18 |
| Keratan sulfate degradation | 3.49E-18 |
| Integrin cell surface interactions | 4.86E-18 |
Fig.S7 Co-expression network of Ptgds obtained from expression data of 31.499 RNA-seq samples (Deelen et al., 2019a) reveals a role for Ptgds in networks involved in elastic fiber formation, resting membrane potential and glutamate binding and activation. Z-scores indicate REACTOME pathway enrichments for the indicated pathways. Also shown are the associated pathways for each cluster.

## Slide 8
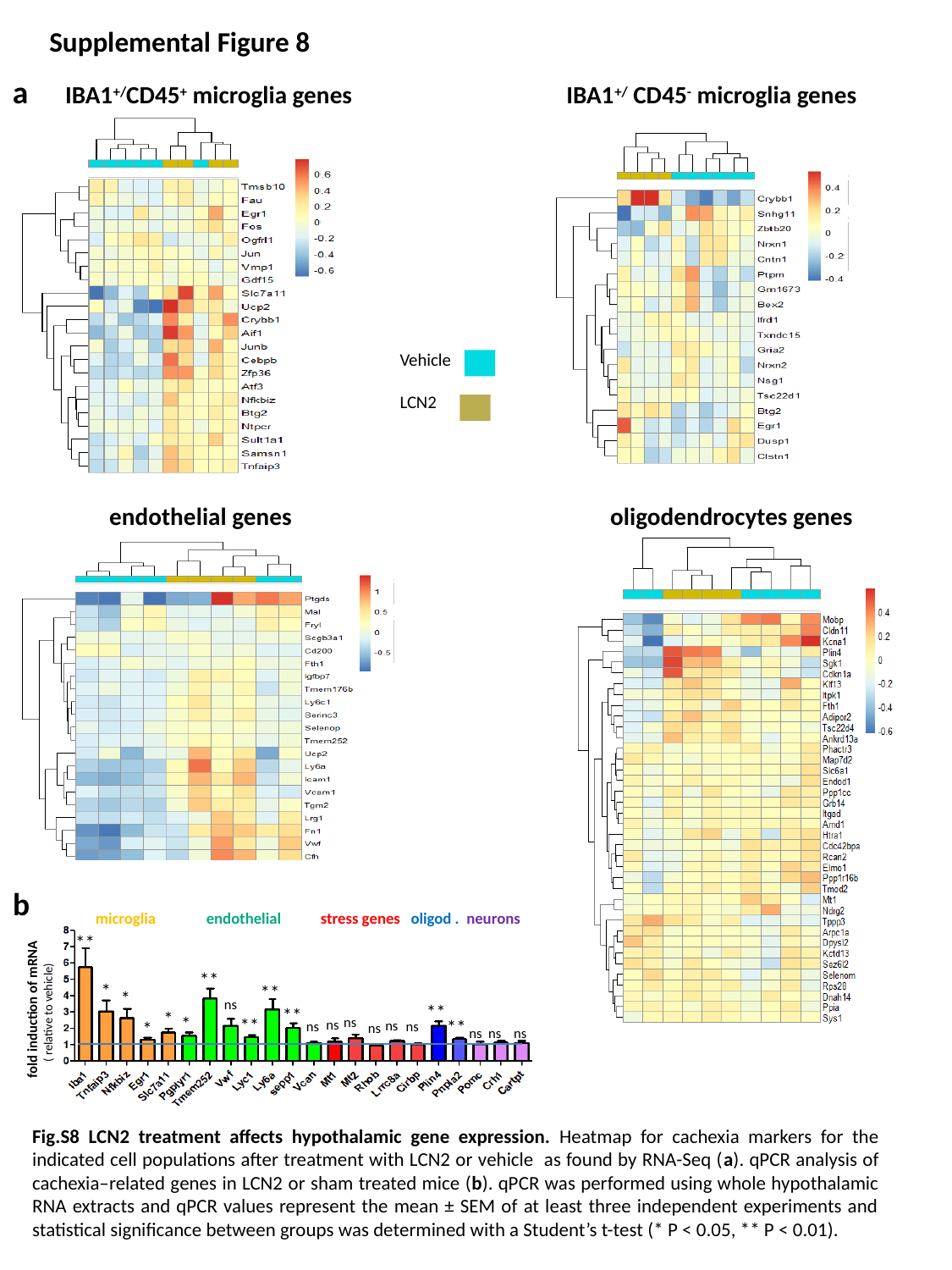

Supplemental Figure 8
a
IBA1+/CD45+ microglia genes IBA1+/ CD45- microglia genes
Vehicle
LCN2
endothelial genes oligodendrocytes genes
b
microglia endothelial stress genes oligod . neurons
**
fold induction of mRNA
 ( relative to vehicle)
**
*
**
*
ns
**
**
*
*
**
ns
**
ns
*
ns
ns
ns
ns
ns ns ns
Fig.S8 LCN2 treatment affects hypothalamic gene expression. Heatmap for cachexia markers for the indicated cell populations after treatment with LCN2 or vehicle as found by RNA-Seq (a). qPCR analysis of cachexia–related genes in LCN2 or sham treated mice (b). qPCR was performed using whole hypothalamic RNA extracts and qPCR values represent the mean ± SEM of at least three independent experiments and statistical significance between groups was determined with a Student’s t-test (* P < 0.05, ** P < 0.01).
