# Supplementary material for: Critical changes in hypothalamic gene networks in response to pancreatic cancer as found by single-cell RNA sequencing
Source: Mol Metab. 2022 Jan 11;58:101441. doi: 10.1016/j.molmet.2022.101441 (PMC8851272; doi:10.1016/j.molmet.2022.101441)
Supplement: Multimedia component 2 [file mmc2.docx]

|  | **qPCR primers** |
| --- | --- |
| *Egr1* | Egr1-Fw TTCAATCCTCAAGGGGAGCC  Egr1-Rv TAACTCGTCTCCACCATCGC |
| *Lrg1* | Lrg1-Fw GCAGCTATGGTCTCTTGGCA  Lrg1-Rv CCTCAGCCGACTGCAGTATC |
| *Mt1* | Mt1-Fw CTCCGTAGCTCCAGCTTCAC  Mt1-Rv CACTTGCAGTTCTTGCAGGC |
| *Mt2* | Mt2-Fw CTCGCCATGGACCCCAAC  Mt2-Rv CAGCCCTGGGAGCACTTC |
| *Aldoc* | Aldoc-Fw AGAAGGAGTTGTCGGATATTGCT  Aldoc-Rv TTCTCCACCCCAATTTGGCTC |
| *Tmem252* | Tmem252-fw AGATGTTCAACCACGTGCTCA  Tmem252-Rv AGGCCTGTTTCTCAACATCCA |
| *Sepp1* | Sepp1-Fw GAAGCTAGTCCGAAGGGGTT  Sepp1-Rv GGGCTTTGTAACAAGCAGAGC |
| *Crybb1* | Crybb1-Fw GCAATCTGCCTTCCGTGGAG  Crybb1-Rv CCATCCTGATGGGCCGGAA |
| *Tnfaip3* | Tnfaip3-Fw CCACTTGGGCTCTGCGAGG  Tnfaip3-Rv ACAGCTTTCCGCATATTGCTCA |
| *Gapdh* | Gapdh- Fw CGACTTCAACAGCAACTCCCACTCTTCC  Gapdh-Rv TGGGTGGTCCAGGGTTTCTTACTCCTT |
| *Pglyrp1* | Pglyrp1-Fw TACCACAAGAATGAGCTGGGC  pglyrp-Rv CCTGTGTGGTCACCCTTGAT |
| *Nfkbiz* | Nfkbiz-Fw TCTCACTTCGTGACATCACC  Nfkbiz-Rv GGTTGGTATTTCTGAGGTGGAG |
| *Scgb3a1* | Scgb3a1-Fw ATAGGTCCTGGGAGCATCTTCT  Scgb3a1-Rv GCAGGCTTGGCCAATGAGTC |
| *Vwf* | Vwf-Fw CAAGGGCTGGAGTGTGCTAA  Vwf-Rv ACACACTTGTTTTCGTGCCG |
| *Tmem176b* | Tmem176b-Fw CTGGAACCTCAAGGGAAGGT  Tmem176b-Rv CACTGTGCTCTGGACCATCTT |
| *Cirpb* | Cirpb-Fw GCCTTAGGAAGCTTGGGTGT  Cirpb-Rv TTGGTGTCGAAGCTGAGTCC |
| *Rhob* | Rhob-Fw CGAGAACTATGTGGCGGACA  Rhob-Rv CTGTCCACCGAGAAGCACAT |
| *Pnpla2* | Pnpla2-Fw CTGACTCGTGTTTCAGACGGA  Pnpla2-Rv TGAAATGCCGCCATCCACAT |
| *Wnt5a* | Wnt5a-Fw2 CGTGGTGTGAATGAACTGGG  Wnt5a-Rv2 GGCGTGATTGTGCAAAAGACT |
| *Crhr1* | Crhr1-Fw GCCCTGCCTTTTTCTACGGT  Crhr1-Rv TCGTTGAGAATCTCCTGGCAC |
| *Plin4* | Plin4-Fw ATGCCCTGAGCCACATACAG  Plin4-Rv CTACCAACAGCCTCCACCAT |
| *Ly6a* | Ly6a-Fw TTATCTGTGCAGCCCTTCTCTG  Ly6a-Rv AGCACTGGTAACACTCCAGTC |
| *Ly6c1* | Ly6c1-Fw CCTGCAACCTTGTCTGAGAGG  Ly6c1-Rv GGCACTCCATAGCACTCGTAG |
| *Igfbp7* | Igfbp7-Fw TCTGATATGGGACACCCAACC  Igfbp7-Rv AAGAACACCTTGGCACCAGT |
| *Cartpt* | Cartpt-Fw GCTACCTTTGCTGGGTGCC  Cartpt-Rv GCTTCGATCTGCAACATAGCG |
| *Gdf15* | Gdf15-2 Fw GAGCTACGGGGTCGCTTC  Gdf15-2-Rv GGGACCCCAATCTCACCT |
| *Iba1* | Iba1-Fw GTCCTTGAAGCGAATGCTGG  Iba-Rv CATTCTCAAGATGGCAGATC |
| *Pomc* | Pomc Fw GGGCGAGCTGATGACCT  Pomc Rv CCGACTGTGAAATCTGAAAGG |
| *Lrrc8a* | LRRC8A-Fw GGGTTGAACCATGATTCCGGTGAC  LRRC8A-Rv GAAGACGGCAATCATCAGCATGAC |
| *Lcn2* | Lcn2-Fw TGGCCACTTGCACATTGTAG  Lcn2-Rv ATGTCACCTCCATCCTGGTC |
| *Nrxn1* | Nrxn1 Fw CTTCCGTGGCAAGGACTGC  Nrxn1-Rv TCAGATCCCTTGAACGTGGC |
| *Slc7a11* | Slc7a11-Fw GGGCCCATAATCAACGGAGAG  Slc7a11-Rv CACGGATCTCCAGGGCTTCT |
| *Vcan* | Vcan-Fw ACCTACACTTGCAAGAAGGGA  Vcan-Rv TGGAAGGTGTCGCTGAATGA |
|  |  |
|  | **In situ hybridization primers** |
| *Ptgds* ISH | Ptgds-EcorI-Fw ATGAATTC CTCGCCTCCAACTCAAGCTG  Ptgds-HINDIII-Rv GAGAAGCTTGCCCTCTGACTGACTTCTCTC |
| *Aldoc* ISH | Aldoc-EcorI-Fw ATGAATTC TGGGGCTGCTACTGAGGAG  Aldoc-HINDIII-Rv TATAAGCTTGAGTTCACGTCCCCCTGATC |
| *Mt1* ISH | Mt1-EcorI-Fw ATGAATTCCGTCACCACGACTTCAAcg  Mt1-HINDIII-Rv TATAAGCTTctCGGTAGAAAACGGGGGTTTAG |
| *Sepp1* ISH | Sepp1-EcorI-Fw ATGAATTCACAGGACGAAGCTAGTCCGAAG  Sepp1-HINDIII-Rv TATAAGCTTTGGTGGCTATGAGCCTCTGAG |
| *Plin4* ISH | Plin4-Fw GCTGACACCAAAACCCTTGT  Plin4-Rv TCTGGTCACTGCACAGCTTC |
